# Spontaneous Closure of the Ductus Arteriosus in Preterm Infants: A Systematic Review

**DOI:** 10.3389/fped.2020.00541

**Published:** 2020-09-11

**Authors:** Johan C. A. de Klerk, Aline G. J. Engbers, Floor van Beek, Robert B. Flint, Irwin K. M. Reiss, Swantje Völler, Sinno H. P. Simons

**Affiliations:** ^1^Division of Neonatology, Department of Pediatrics, Erasmus UMC—Sophia Children's Hospital, Rotterdam, Netherlands; ^2^Division of Systems Biomedicine and Pharmacology, Leiden Amsterdam Center for Drug Research (LACDR), Leiden University, Leiden, Netherlands; ^3^Department of Hospital Pharmacy, Erasmus UMC, Rotterdam, Netherlands; ^4^Division of BioTherapeutics, Leiden Amsterdam Center for Drug Research (LACDR), Leiden University, Leiden, Netherlands

**Keywords:** patent ductus arteriosus, spontaneous closure, preterm infants, systematic review, VLBW, ELBW

## Abstract

The optimal management strategy for patent ductus arteriosus in preterm infants remains a topic of debate. Available evidence for a treatment strategy might be biased by the delayed spontaneous closure of the ductus arteriosus in preterm infants, which appears to depend on patient characteristics. We performed a systematic review of all literature on PDA studies to collect patient characteristics and reported numbers of patients with a ductus arteriosus and spontaneous closure. Spontaneous closure rates showed a high variability but were lowest in studies that only included preterm infants with gestational ages below 28 weeks or birth weights below 1,000 g (34% on day 4; 41% on day 7) compared to studies that also included infants with higher gestational ages or higher birth weights (up to 55% on day 3 and 78% on day 7). The probability of spontaneous closure of the ductus arteriosus keeps increasing until at least 1 week after birth which favors delayed treatment of only those infants that do not show spontaneous closure. Better prediction of the spontaneous closure of the ductus arteriosus in the individual newborn is a key factor to find the optimal management strategy for PDA in preterm infants.

## Introduction

After preterm birth, the ductus arteriosus often remains open. Patent ductus arteriosus (PDA) in preterm infants has been associated with prolonged ventilation, bronchopulmonary dysplasia (BPD), and necrotizing enterocolitis potentially caused by pulmonary overcirculation and systemic hypoperfusion (1). It is unclear if these associations reflect a causal relationship or if PDA is a marker of poor condition and outcome, because outcomes of well-designed and controlled trials are still awaited (2). Treatment options include fluid restriction, pharmacological intervention with non-steroidal anti-inflammatory drugs (NSAIDs) or paracetamol, or closing the duct by surgical ligation or heart catheterization. All of these therapeutic options have their side-effects or specific risks in preterm infants. As a consequence, there is an ongoing worldwide discussion about the optimal management of PDA in preterm infants (3). This discussion is complicated by the lack of extensive knowledge on the (patho)physiology of the ductus arteriosus in preterm infants.

Intrauterine, the ductus arteriosus is needed and remains open due to the hypoxic fetal environment and by prostaglandins E2 (PGE2) produced by the placenta (4). Vasodilatation is further enhanced by nitric oxide (NO) produced by the wall of the ductus arteriosus (5). Upon term birth, the ductus arteriosus normally closes within hours. This is the result of different complex physiologic mechanisms that include changes in pulmonary and systemic vascular resistance, increase in arterial oxygen pressure, decreasing levels of prostaglandins and changes in different mediators and growth factors (6). After preterm birth, however, the ductus arteriosus frequently remains patent. Even after functional closure of the ductus arteriosus, either spontaneous or by pharmacological treatment, it might re-open in preterm infants afterwards caused by infection or increased inflammation (7).

Although the high levels of prostaglandins produced by the placenta also drop after preterm birth, the ductus arteriosus seems to remain much more sensitive to both PGE2 and NO in preterm infants compared to term born infants, due to increased expression of—and binding to—prostaglandin receptors in the ductal wall (5, 8). In addition to that, oxygenation and vascular resistance after preterm birth are hampered by an immature cardiovascular system and insufficient breathing whereas oxygen targets are decreased to prevent retinopathy of prematurity (9).

A substantial part of the preterm infants with PDA still seems to show delayed ductus closure without any intervention (10). The advantage of a wait-and-see PDA treatment strategy above intervention in the 1st days after birth (<72 h) may be that only those newborns without spontaneous ductus arteriosus closure are exposed to treatment and its potential side effects. On the other hand, early treatment strategy may be favored because the pharmacological closure rate seems to be highest on the 1st days of life (11, 12). Despite the limited studies on delayed treatment with NSAIDs and paracetamol, this late treatment seems less effective (13, 14). NSAIDs and paracetamol may enhance the spontaneous closure process. Therefore, the discussion on management of PDA in preterm infants cannot neglect the spontaneous closure, although a clear overview of the spontaneous closure rates is yet lacking. In this study, we aimed to provide such an overview of all available data from PDA studies to investigate and analyze the rates of spontaneous closure of the PDA.

## Methods

To retrieve all relevant evidence on the physiological spontaneous ductus arteriosus closure in preterm infants, we performed a thorough literature search. All studies published after 1990 that met both of the following criteria were considered eligible: (1) trials of any form including randomized controlled trials (RCTs), controlled clinical trials, quasi experimental studies [(un)controlled before and after studies], prospective and retrospective cohort studies and case-control studies and (2) trials with a clearly described timing of echocardiography to identify the presence or absence of a ductus arteriosus. Case series and case reports were excluded, as well as studies that reported on spontaneous closure after discharge. To examine spontaneous closure of the PDA, data on spontaneous closure before any intervention were collected and analyzed.

### Search Strategy

A literature search was performed in collaboration with an experienced librarian. The search was done in MEDLINE, EMBASE, Cochrane central, Web of science, and Google Scholar until 2018 and included only English written articles. The following search terms were used “patent ductus arteriosus,” “PDA,” “preterm,” “VLBW,” and/or “prematurity.” A more detailed search strategy for each library is available in Supplementary File 1.

The retrieved titles, abstracts, and full text were screened by two independent reviewers (JdK and FvB) to assess their eligibility according to pre-established criteria. Duplicate publications were excluded. The data extraction was done by the same two independent reviewers (JdK and FvB). Discrepancies were either resolved by discussion or by consulting a third reviewer (SS).

### Data Synthesis

We developed a data extraction sheet, pilot-tested it on 10 randomly-selected included studies, and refined it accordingly. One review author (JdK) extracted the following data from included studies and the second author (FvB) checked the extracted data. Disagreements were resolved by discussion between the two review authors; if no agreement could be reached, it was planned a third author (SS) would decide. The data that was used included the following: total number of included neonates, their gestational age and birth weight, timing of the echocardiographic evaluation, and the number of neonates with closed ductus arteriosus at those times. Because only baseline reports of the occurrence of PDA were included, before any intervention for PDA was initiated, no bias of individual studies was expected.

Articles were categorized based on the inclusion criteria that were used in the studies for gestational age (GA) and birth weight (BW). Based on frequently used inclusion cut-off values of GA and BW, four different groups were defined prior to data collection: group 1: GA < 28 weeks and/or BW < 1,000 g, group 2: GA < 30 weeks and/or BW < 1,250 g, group 3: GA < 32 weeks and/or BW < 1,500 g, and group 4: GA < 37 weeks and/or BW < 2,500 g. If only GA or BW was given as inclusion criterion, this determined the category of the article, if both GA and BW were given as inclusion criteria, GA was leading for categorization. Studies were only included in one group: those included in group 1 were not included in groups 2–4, studies in group 2 were not included in group 3 and 4, and studies in group 3 were not included in group 4.

### Data Analysis

The primary outcome of this systematic review was the rate of spontaneous closure of the ductus arteriosus in preterm infants as evaluated by echocardiography. A closed ductus arteriosus is defined as a ductus arteriosus that shows complete closure or no doppler flow on echocardiography as reported in the original reports. The closure rate was calculated as the part of patients with a closed ductus within a certain cohort (number of patients with a closed ductus divided by the total number of patients) at a time-point. To further explore how the observed trends were correlated to the maturational status of the patient, different subgroup analyses were performed for the different GA and BW groups.

R Software (V 3.5.1) was used in R Studio (V 1.1.643) to group, summarize, and visualize the data. The percentage of patients with PDA was plotted against postnatal age. To differentiate spontaneous closure between the different GA and BW groups, a linear smoothed line weighted by the total number of patients of each study was drawn. Mean percentages of patients with PDA of studies that performed an echocardiography on postnatal day 3 (between 72 and 95.9 h) and day 7 (between 168 and 191.9 h) were calculated, and were weighted by the number of patients in each study. These time-points were included because the ductus is mostly evaluated during the 1st days of life (<72 h) and administered courses of pharmacotherapy normally take 3 or 6 days.

## Results

**Figure 1:**
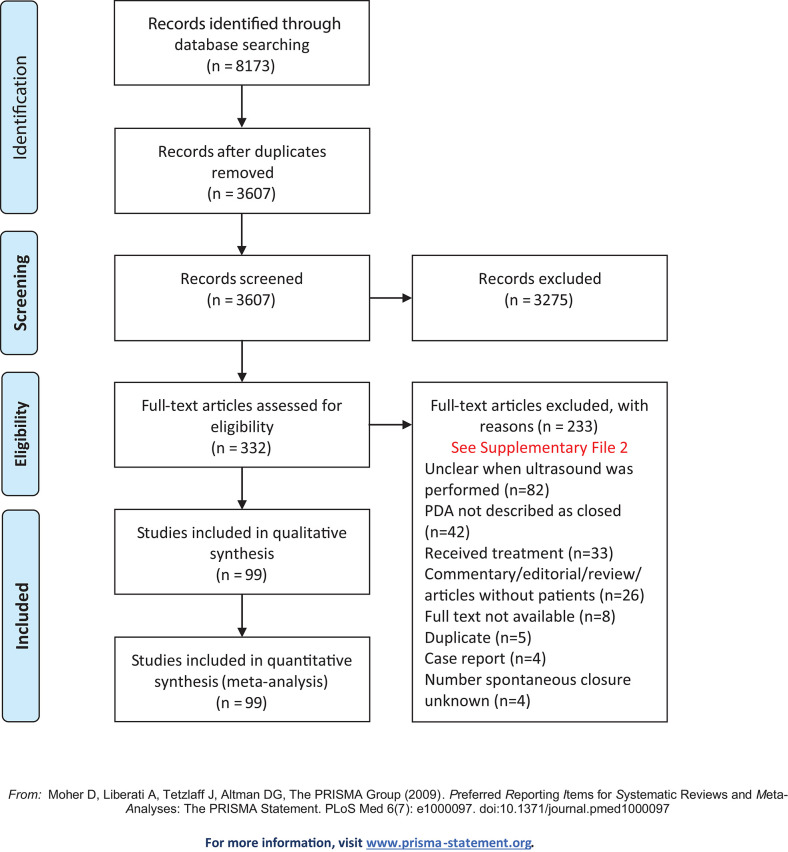
Flow diagram of the studies retrieved for this review.

**Figure 2:**
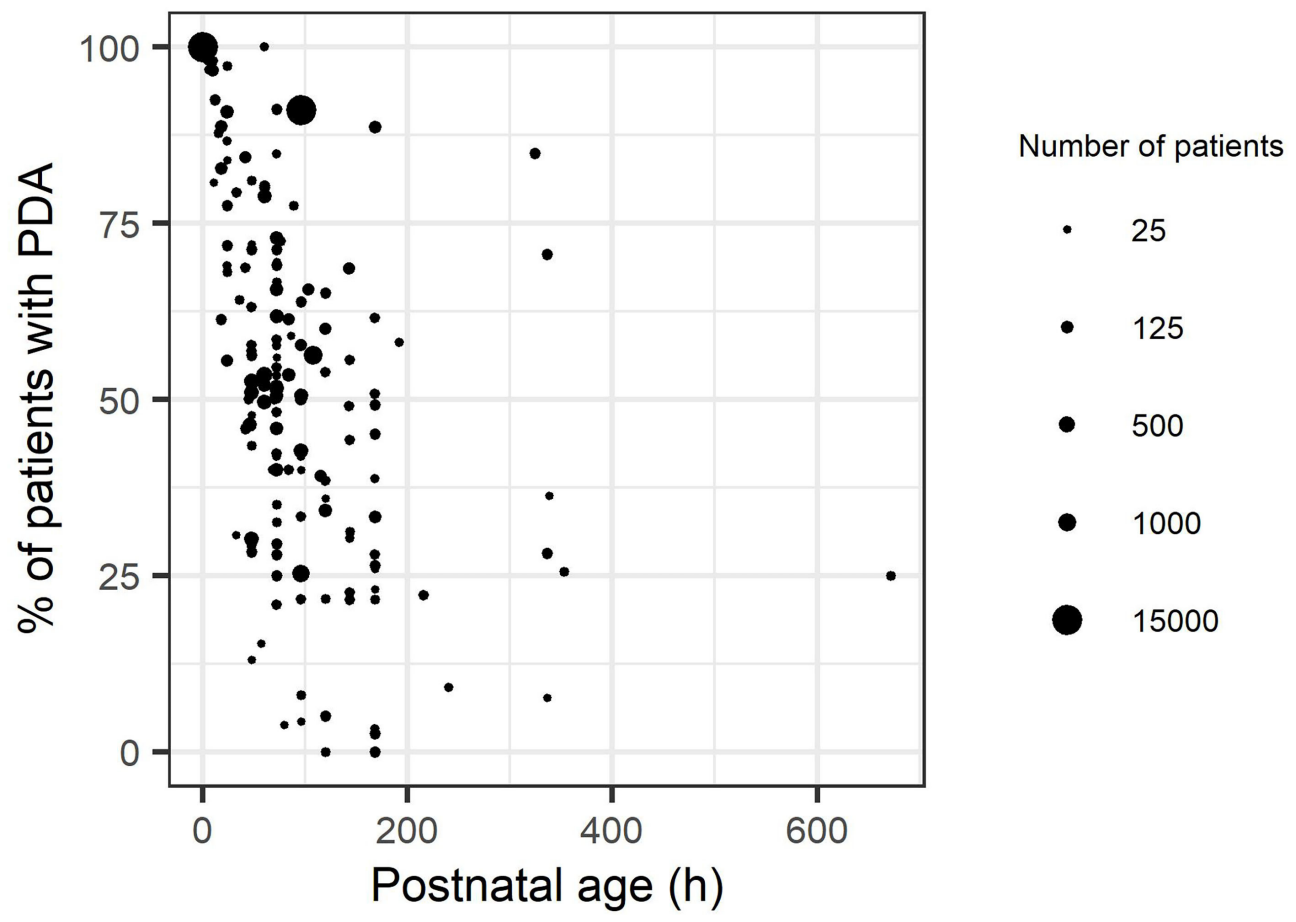
Reported percentages of closure of patent ductus arteriosus of all included studies, up to a postnatal age of 28 days. Each dot represents a reported percentage, whose size represents the square root of the total number of patients of that observation.

Our literature search resulted in a retrieval of 8,173 records. After removing the duplicates 3,607 remained. Reading of the titles and abstracts resulted in 332 eligible articles. The arguments for exclusion of 233 articles after full text screening are listed in Supplementary File 2. The clinical characteristics of the included studies are summarized in Supplementary File 3. All studies were published between 1990 and 2018. Ninety-nine articles with a total of 29,532 patients were included in the analysis. In Figure 1, a flow diagram is presented of the studies retrieved for this review. Figure 2 presents the reported percentages of patients with a PDA for each individual study with increasing postnatal ages. On PNA day 3 (all reported outcomes observed between 72 and 96 h of life), the mean reported percentage of patients that experienced spontaneous closure of the ductus arteriosus weighted by the number of patients was 47% (range 9–96%). On PNA day 7 (168–192 h) this percentage increased to 61% (11–100%).

### Subgroup Analysis

In Table 1, the reported characteristics of all studies are presented per group, as well as the percentages of PDA closure at postnatal age days 3 and 7 and the studies and number of patients these are based on.

**Table 1:** Summary of reported mean or median gestational age and birthweight of the included studies, and reported percentages of PDA closure at postnatal age days 3 and 7.

	**Group 1**	**Group 2**	**Group 3**	**Group 4**	**All studies**
Inclusion criteria	GA < 28 weeks and/or birth weight <1,000 g	GA < 30 weeks and/or birth weight <1,250 g	GA < 32 weeks and/or birth weight <1,500 g	GA < 37 weeks and/or birth weight <2,500 g	
Number of studies	11	20	49	19	99
Total number of patients	17,156	2,980	6,946	2,450	29,532
**Gestational Age**					
Mean (weeks) [median (range)] (*n*)	26.0 [25.5–26.6] (8)	28.0 [26.2–28.8] (13)	28.4 [26.0–30.0] (22)	30.8 [30.2–31.1] (7)	28.1 [25.5–31.2] (50)
Median [median (range)]	–	28.0 [–] (2)	29 [27–31] (15)	31.0 [28.1–31.0] (5)	29.0 [27.0–31.0] (22)
Not reported (*n*)	3	5	12	7	27
**Birth weight**					
Mean [median (range)]	818 [802–851] (8)	1,028 [(797–1,259] (13)	112 [794–1,371] (22)	1,543 [1,355–1,917] (7)	1,082 [794–1,917] (50)
Median [median (range)]	–	1,060–1,062 (2)	1,160 [980–1,595] (15)	1,475 [950–1,640] (5)	1,160 [950–1,640] (22)
Not reported (*n*)	3	5	12	7	27
**Postnatal age of cardiac ultrasound**					
Median postnatal age in h (range)	72 (18–1,464)	72 (5–672)	79 (6–3,864)	72 (24–1,632	72 (5–3,864)
**Percentage of PDA closure at postnatal age 3 (72–95.9 h)**					
Weighted mean percentage of patients with PDA (range)	34% (9–71)	47% (33–98)	48% (22–65)	55% (15–79)	47% (9–96)
Number of studies with reported percentage	6	9	16	8	39
Total number of patients	646	978	1,709	621	3,954
Study references	(15–20)	(21–29)	(30–45)	(46–53)	(15–53)
**Percentage of PDA closure at postnatal age 7 (168–191.9 h)**					
Weighted mean percentage of patients with PDA (range)	41% (11–97)	77 % (–)	63% (38–100)	78% (67–97)	61% (11–100)
Number of studies with reported percentage	2	1	8	5	16
Total number of patients	228	26	550	181	985
Study references	(18, 54)	(24)	(30, 33, 43, 55–59)	(46, 50, 60–62)	(18, 24, 30, 33, 43, 46, 50, 54–62)

*Separated by groups based on inclusion criteria*.

Eleven different articles were included in group 1 which contained preterm infants with a gestational age under 28 weeks and/or birth weight under 1,000 g (15–20, 54, 63–66). The median of reported mean or median GAs of the 17,156 patients in group 1 was 26.0 (range of mean/median 25.5–26.6) weeks. The median birth weight was 832 (range of medians 802–851) g. Exact numbers for gestational ages and birth weights were not available for three studies (19, 63, 66). Two of the 11 studies performed multiple cardiac ultrasounds to evaluate the PDA, ranging from 24 h until 61 days of postnatal age (15, 18). Seven of the 11 studies performed their first ultrasound at day 3 after birth.

Twenty articles were included in group 2 (GA < 30 weeks and/or BW < 1,250 g) (11, 21–29, 67–76). The median of the reported mean or median GA of the 2,980 patients in group 2 was 28.0 (range 26.2–28.8). The median of the reported mean or median birth weight was 1,028 (range 797–1,259) g. The exact gestational ages and birth weights were not available for four studies (28, 29, 69, 74). Seven of the 20 studies performed multiple cardiac ultrasounds to evaluate the PDA (21, 24, 25, 27, 67, 72, 73). The echocardiography was performed between 5 h and 28 days of postnatal age in all studies.

Forty-nine studies that included a total of 6,946 patients were eligible for group 3 (GA < 32 weeks and/or BW < 1,500 g) (30–45, 55–59, 77–103, 132). The median GA was 28.6 (range 26–31) weeks. The median birth weight was 1,120 (range 794–1,595) g. Exact gestational ages and birth weights were not available for nine studies (35, 38, 41, 42, 44, 85, 96, 100, 104). Multiple cardiac ultrasounds where performed in 14 of the 49 studies and were performed between 6 and 338 h of postnatal age.

Nineteen studies used GA < 37 weeks and/or birthweight < 2,500 g as inclusion criteria (group 4), in which a total of 2,450 patients were included (46–53, 60–62, 105–112). The median gestational age was 30.9 (range 28.1–31.2) weeks. The median birth weight was 1,479 (range 950–1,917) g. Gestational ages and/or birth weights were not available for six studies (47, 50, 53, 105, 107, 109). Six of the 19 studies performed multiple cardiac ultrasounds at varying post-natal ages between 24 and 168 h.

**Figure 3:**
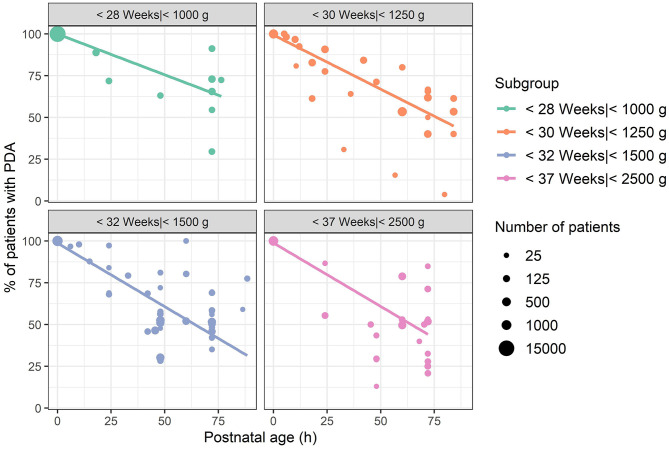
Reported percentages of patients with patent ductus arteriosus up to a postnatal age of 4 days, grouped by mean or median gestational age or birthweight if gestational age was unreported. Each dot represents one observation at the reported postnatal age. The size of the dots represents square root of the number of patients of each observation. Lines represent a linear smooth weighted by the number of patients of each observation.

**Figure 4:**
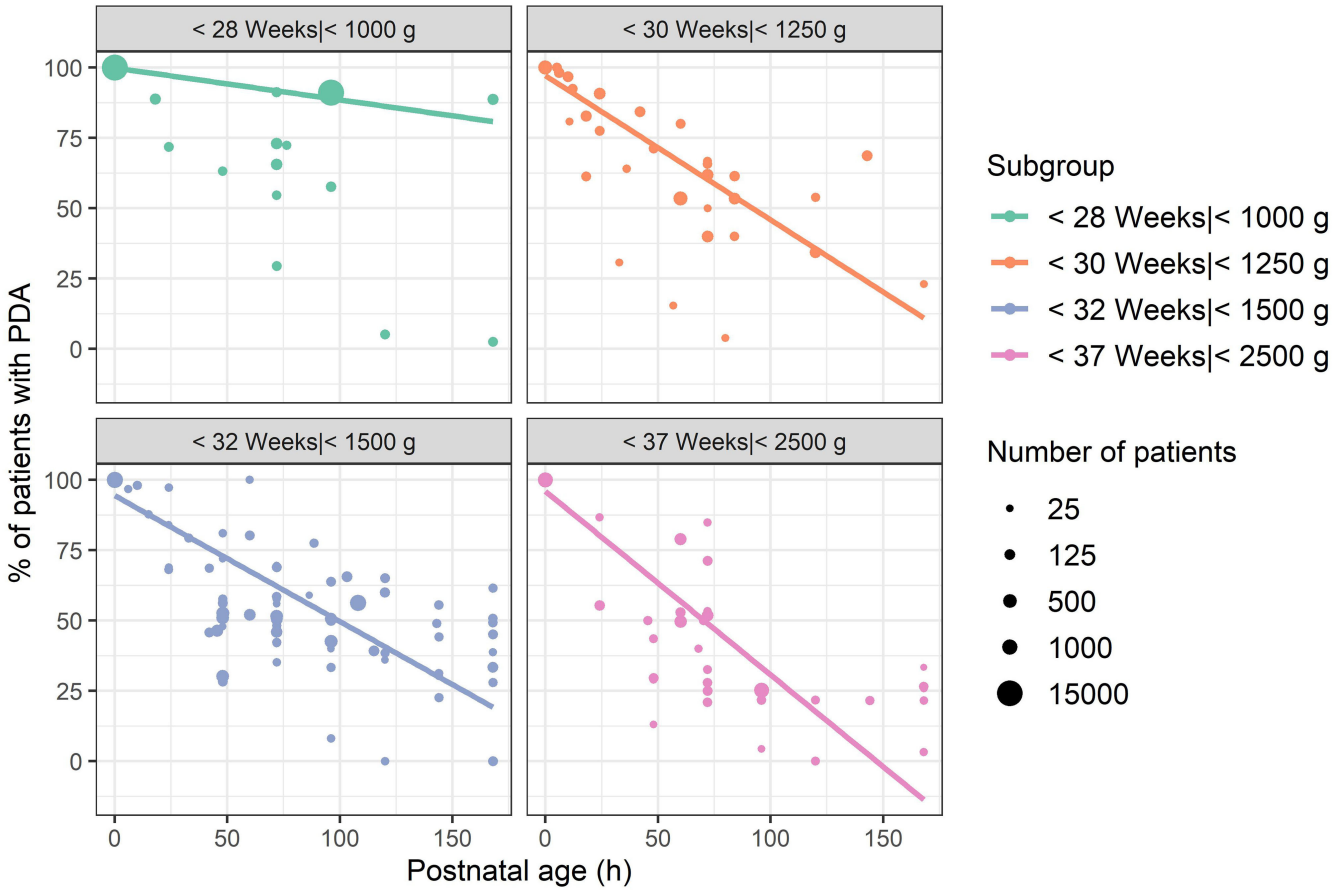
Reported percentages of patients with patent ductus arteriosus up to a postnatal age of 8 days, grouped by mean or median gestational age or birthweight if gestational age was unreported. Each dot represents one observation at the reported postnatal age. The size of the dots represents square root of the number of patients of each observation. Lines represent a linear smooth weighted by the number of patients of each observation.

In Figures 3 and 4 the reported percentage of preterm infants with PDA are presented for each subgroup with a postnatal age up to 3 and 7 days, respectively. To account for the large differences in number of patients (range 18–15,971), the size of the dots is scaled by the square root of the number of patients. At postnatal age day 3 (72–95.9 h), mean percentage of PDA closure was 34% for group 1, weighted by the number of patients in each study (range 9–71%). In group 2, this percentage was 47% (33–98%), and in group 3 this was 48% (22–65%). In group 4, the weighted mean was 55% at PNA 3 (15–97%).

At PNA 7, the weighted average of patients with a closed PDA was 41% for group 1 (11–97%). Of group 2, only one study was available with a reported percentage of closure at PNA 7, which was 77% (20 of 26 patients) (24). In group 3, the weighted average closure was 63% (38–100%) at PNA day 7, and for group 4 this was 78% (67–97%). Comparing Figures 2–4 clearly show that with increasing postnatal ages the spontaneous closure continuous and PDA rates decrease. This is most obvious in the studies that also included the oldest groups of infants.

## Discussion

In this review, spontaneous closure rates of the ductus arteriosus in preterm neonates were systematically evaluated in 99 studies that represented 29,532 patients. As expected, we observed increasing rates of ductus closure with post-natal age and higher spontaneous closure rates in studies that also included patients with higher gestational ages. Spontaneous closure, however, occurs not only in the 1st day of life, but continues throughout the 1st week of life. Our systematic review revealed 34% spontaneous closure on the 3rd day of life (72–96 h) in the studies that only included the youngest group of infants (<28 weeks of GA and/or birthweight <1,000 g). If older infants were also included in studies, these closure rates increased up to 55%. At PNA day 7 (168–192 h) the ductus arteriosus was closed in 41% of the newborns in studies of the youngest infants and up to 78% in the studies that also included older gestational age groups. Because of a lack of detailed reports in the individual studies on subgroups of patients and the lack of longitudinal assessment of the ductus arteriosus we were unable to provide a mathematical function of spontaneous ductus closure in preterm infants. Such a function that could predict spontaneous closure in an individual patient would be an ultimate goal to guide PDA management in individual patients. High quality datasets with repeated echocardiographic assessments of neonatal patients treated in current neonatal intensive care units are needed first.

Studies that reported on PDA showed large heterogeneity, not only in included patient populations, but also in the definition of a hemodynamically significant PDA (hsPDA) (113). It is quite clear that the significance of a PDA is not only determined by the diameter of the ductus, as was used in the current review, but also by the pulmonary vascular resistance. The lack of consensus on the definition of a hsPDA is partly based on the lack of validated echocardiographic markers and cutoff values. van Laere et al. (114) proposed to standardize essential echocardiographic measurements for the assessment of hemodynamic significance of a PDA. These consist of evaluation of the ductus arteriosus itself (including the diameter, flow direction, and velocity), indices of pulmonary overcirculation (La:Ao, left pulmonary artery diastolic flow) and indices of systemic shunt effect (flow pattern in aorta descendens, tructus coeliacus, or middle cerebral artery) (114). The LA:Ao ratio, ductal diameter and diastolic flow in the left pulmonary artery are easy to measure and seem the most accurate and easy to determine markers for a hsPDA (115). In our aim to select those infants that would not show spontaneous closure and might actually need PDA treatment these markers would be useful.

Currently, there is no international consensus on PDA management. It is unclear if, how and when PDA in preterm infants should be treated. More specifically, it is unclear if PDA needs treatment because it is unknown which preterm infants might benefit more from treatment than others. Therefore, prophylaxis, early treatment (<24 h), late treatment (72 h), symptomatic treatment and wait and see strategies are currently used alongside each other (116). Better knowledge on the spontaneous closure and physiology of the ductus arteriosus in preterm infants may help to determine the optimal management strategy. This is even more important as prophylactic as well as therapeutic treatment strategies are associated with risks for adverse effects, such as intraventricular hemorrhages and decreased renal function that might have severe consequences in these vulnerable patients. Treatment of the PDA, pharmacologically or surgically, should therefore be reserved for those patients who may benefit from it.

Next to the ongoing discussion on the type of drug being either ibuprofen, indomethacin, or acetaminophen (117–124), the timing of treatment initiation varies widely between studies, and might explain reported differences in efficacy. As spontaneous ductus closure increases with PNA, part of ductus closure reported in prophylactic and early treatment studies may be due to spontaneous closure rather than drug treatment. Efficacy of PDA pharmacotherapy seems to decrease, with post-natal age, even if dosages are increased with PNA to correct for increased clearance with age (10, 13). This suggests either a certain window of opportunity for PDA treatment during the physiological process that is involved in the spontaneous closure of the ductus arteriosus or the need for higher drug exposures at older ages. Such a window can only be identified if the rate of spontaneous closure is well-characterized, and efficacy studies can correct for the chance of spontaneous closure.

With the current review, we have summarized the evidence that spontaneous closure is less likely to occur in preterm neonates with the lowest gestational ages. Nevertheless, in another significant number of preterm infants the ductus arteriosus although delayed, closes spontaneously within the first 150 h of life. The presented review was limited by the lack of detailed reports of PDA for different gestational age groups in the included studies. While the literature was systematically reviewed we were unfortunately unable to provide statistical analysis due to complexity of outcome data. This also resulted in overlap of patients in the different groups. All studies only had an upper limit of gestational age and/or bodyweight without a lower limit for gestational age/weight. As a consequence, the gestational age/weight range of inclusion criteria of the included studies increases from group 1 to 4. Therefore, the actual difference in the occurrence of spontaneous closure between different GA groups is bigger than observed in the present study. As other widely used measures for ductus closure, such as LA:Ao ratio were mostly missing, we defined a closed ductus as a ductus that was visually closed or when there was no doppler flow visible on echocardiography. As discussed previously, a better definition of a hsPDA might be desirable and might lead to a more precise estimation of incidence- and closure-rates. In this study, we presented the relationships between spontaneous closure rate and PNA as linear relationships. In reality, the spontaneous closure rate is probably highest on the 1st day and decreases with an asymptotic shape since in some patients the spontaneous closure will not occur, or the percentage of patients with PDA might even go up if the ductus arteriosus reopens. With the available data, it is not possible to determine whether the reported numbers of PDA at high postnatal ages (Figure 2) are due to non-closure or re-opening because seventy of the 99 articles performed only one echocardiography. Therefore, there were insufficient data to study potential reopening of the ductus arteriosus in this review. The patency of the ductus arteriosus is regulated by the balance of vasodilating (prostaglandin E2, nitric oxide) and vasoconstrictor (oxygen) factors (6). Preterm neonates are more sensitive to the vasodilating factors compared to the term neonates (5). There is some evidence suggesting that genetic variations may play a role in the occurrence of PDA in preterm infants. In a large cohort of 1,013 preterm neonates Dagle et al. (125) found that several single nucleotide polymorphisms that were associated with PDA. For future meta-analyses, it might thus be of interest to include genetic variations to determine their influence on spontaneous closure.

Our systematic review was based on the assumption that gestational age is a more important factor compared to birthweight for patent ductus arteriosus in preterm infants. Villamor-Martinez et al. (126) showed that small for gestational age (SGA) infants showed a significantly reduced risk of PDA, but their review was complicated by the heterogeneity of studies. As SGA infants also show a much higher clearance of ibuprofen compared to appropriate weight for age newborns these patients form a special population that need additional attention in future PDA studies (127).

A next step could be to find markers to repeatedly monitor closure and pathophysiology of the ductus arteriosus. These could include the continuously available perfusion index to identify a hemodynamic significant PDA (51, 128) or urinary prostaglandine levels (129). Neutrophil gelatinase-associated lipocalin and heart-type fatty acid-binding protein are two peptides that can be measured in urine and also appear to be promising future markers to quantify the effect of a PDA on systemic perfusion, which makes the ductus arteriosus more hemodynamic significant (130, 131). Relevant risk factors could help to predict those patients whose ductus arteriosus will remain open and for whom pharmacological treatment might be needed.

Spontaneous closure rates increase with both gestational age and postnatal age. This review showed that in 34% of the most premature infants (GA < 28 weeks and/or BW < 1,000 g), the ductus arteriosus had spontaneously closed on the 3rd day of life (72–96 h). This increased to 41% at PNA day 7. As patients with a GA < 28 weeks have the lowest chance of spontaneous closure of the ductus arteriosus in the 1st days of life, studies on PDA management should therefore focus on these most premature patients.

## Data Availability Statement

The original contributions presented in the study are included in the article's Supplementary Material, further inquiries can be directed to the corresponding author.

## Author Contributions

JK is responsible for the study design, data collection and extraction, and writing and editing the article. AE is responsible for the data analysis and writing the article. SV helped with the analysis and edited the manuscript. FB performed the data collection and extraction. RF supervised the design and edited the manuscript. IR supervised the design and edited the manuscript. SS supervised the study design, data collection, and contributed with the editing of the article. All authors contributed to the article and approved the submitted version.

## Conflict of Interest

The authors declare that the research was conducted in the absence of any commercial or financial relationships that could be construed as a potential conflict of interest.
