# Supplementary material for: Spontaneous Closure of the Ductus Arteriosus in Preterm Infants: A Systematic Review
Source: Front Pediatr. 2020 Sep 11;8:541. doi: 10.3389/fped.2020.00541 (PMC7516116; doi:10.3389/fped.2020.00541)
Supplement: Supplementary file 1 [file Table_1.DOCX]

Supplementary file 1: Electronic database search strategies:

***Embase.com, language English:***

**(**'patent ductus arteriosus'/**mj/exp** OR (((paten*) NEAR/3 (duct*) NEAR/3 (arterios* OR botalli))):ab,ti**) AND (**'prematurity'/de OR (prematur* OR preterm* OR 'pre-term' OR VLBW OR ELBW OR ((24 OR 25 OR 26 OR 27 OR 28 OR 29 OR 30 OR 31 OR 32 OR 33 OR 34 OR 35 OR 36) NEXT/1 (week* OR wks)) OR ((low) NEAR/3 (birth))):ab,ti**) NOT (**'conference abstract'/it**) NOT (**[animals]/lim NOT [humans]/lim**) AND** english:la

***Medline Epub (Ovid):***

(exp *"Ductus Arteriosus, Patent"/ OR (((paten*) ADJ3 (duct*) ADJ3 (arterios* OR botalli))).ab,ti.) **AND** (exp "Infant, Premature"/ OR "Premature Birth"/ OR (prematur* OR preterm* OR "pre-term" OR VLBW OR ELBW OR ((24 OR 25 OR 26 OR 27 OR 28 OR 29 OR 30 OR 31 OR 32 OR 33 OR 34 OR 35 OR 36) ADJ1 (week* OR wks)) OR ((low) ADJ3 (birth))).ab,ti.) **NOT** (abstract).pt. **NOT** ((exp animals/ OR (lamb* OR rat OR rats OR dog* OR cats).ab,ti.) NOT humans/) **AND** English.lg.

***Cochrane Central:***

**(**(((paten*) NEAR/3 (duct*) NEAR/3 (arterios* OR botalli))):ab,ti**) AND (**(prematur* OR preterm* OR 'pre-term' OR VLBW OR ELBW OR ((24 OR 25 OR 26 OR 27 OR 28 OR 29 OR 30 OR 31 OR 32 OR 33 OR 34 OR 35 OR 36) NEXT/1 (week* OR wks)) OR ((low) NEAR/3 (birth))):ab,ti**)**

***Web of Science:***

**TS=((**(((paten*) NEAR/2 (duct*) NEAR/2 (arterios* OR botalli)))**) AND (**(prematur* OR preterm* OR "pre-term" OR VLBW OR ELBW OR ((24 OR 25 OR 26 OR 27 OR 28 OR 29 OR 30 OR 31 OR 32 OR 33 OR 34 OR 35 OR 36) NEAR/1 (week* OR wks)) OR ((low) NEAR/2 (birth)))**) NOT (**(animal* OR lamb* OR rat OR rats OR dog* OR cats) NOT (human* OR patient*)**)**) **AND** DT=Article **AND** LA=English

***Google Scholar:***

"patent|patency duct|ductus arteriosus|botalli**"** premature|preterm|VLBW|ELBW|"low birth"
