# Supplementary material for: Spontaneous Closure of the Ductus Arteriosus in Preterm Infants: A Systematic Review
Source: Front Pediatr. 2020 Sep 11;8:541. doi: 10.3389/fped.2020.00541 (PMC7516116; doi:10.3389/fped.2020.00541)
Supplement: Supplementary file 2 [file Table_2.docx]

Supplementary File 2: List of excluded studies after full text screening

| No. | No. endnote | Study reference | Reason for exclusion |
| --- | --- | --- | --- |
| 1 | 30 | Ramos FG, Rosenfeld CR, Roy L, Koch J and Ramaciotti C. Echocardiographic predictors of symptomatic patent ductus arteriosus in extremely-low-birth-weight preterm neonates. Journal of Perinatology. 2010. 30: 535 – 539 | Duplicate |
| 2 | 46 | Selahattin Akar, Nilgun Karadag, Tulin Gokmen Yildirim, Handan Hakyemez Toptan, Emre Dincer, Abdulhamit Tuten, Taner Yavuz, Sevilay Topcuoglu, Hande Ozgun Karatepe, Elif Ozalkaya, Guner Karatekin, Fahri Ovali. Does platelet mass influence the effectiveness of ibuprofen treatment for patent ductus arteriosus in preterm infants? The journal of maternal-fetal and neonatal medicine. 2016. 23: 3786 – 3789 | Outcome not relevant; hsPDA not definitively closed |
| 3 | 57 | Jong-Hau Hsu, San-Nan Yang, Hsiu-Lin Chen, Hsing-I. Tseng, Zen-Kong Dai, and Jiunn-Ren Wu. B-type natriuretic peptide predicts responses to indomethacin in premature neonates with patent ductus arteriosus. The journal of pediatrics. 2010. 157: 79 - 84 | Only data from treated patients |
| 4 | 58 | Sabry Ghanem, Mansour Mostafa, Mohamed Shafee. Effect of oral ibuprofen on patent ductus arteriosus in premature newborns. Journal of the Saudi Heart Association. 2010. 22: 7 – 12 | Not obvious when ductus closed |
| 5 | 67 | Gregory P Moore, Sarah L Lawrence, Gyaandeo Maharajh, Amanda Sumner, Isabelle Gaboury, Nick Barrowman, Brigitte Lemyre. Therapeutic strategies, including a high surgical ligation rate, for patent ductus arteriosus closure in extremely premature infants in a North American centre. Pediatr Child Health. 2012. 17 (4); e26 – e31. | All received some kind of treatment |
| 6 | 69 | Cheng Han Lee, Hsiao-Neng Chen, Long-Yen Tsao, Chien-Chou Hsiao, Meng-Luen Lee. Oral ibuprofen versus intravenous Indomethacin for closure of patent ductus arteriosus in very low birth weight infants. Pediatrics and neonatology. 2012. 53: 346 – 353. | No information on total group |
| 7 | 73 | Jang Hoon Lee, Moon Sung Park, Jeong Hee Shin, Byung Min Choi. Can early B-type natriuretic peptide assays predict symptomatic patent ductus arteriosus in extremely low birth weight infants. Neonatology. 2013. 103: 118 – 122 | Outcome not relevant: hsPDA not definitively closed |
| 8 | 82 | Satoko Ito, Tadashi Matsuda, Ryuta Kitanishi, Haruo Usuda, Takushi Hanita, Shimpei Watanabe, Tatsuya Watanabe and Osamu Adachi. Surgical ligation for patent ductus arteriosus in extremely premature infants: strategy to reduce their risk of neurodevelopmental impairment. Tohoku J. Exp. Med. 2016. 240: 7 – 13 | No data which day ultrasound was performed |
| 9 | 93 | Fatih Aygün, Nilgün Köksal, Özlem M. Bostan, Fahrettin Uysal,  İpek Güney Varal, Pelin Doğan. A major cause of mortality and morbidity of very low birth weight infants: patent ductus arteriosus. Guncel pediatr. 2012. 1: 8 – 12 | Article in Turkish, while abstract in English was |
| 10 | 116 | Ece Yapakçı, Ayşe Ecevit, Deniz Anuk İnce, Mahmut Gökdemir, M. Agah Tekindal, Hande Gülcan, Aylin Tarcan. Inferior vena cava oxygen saturation during the first three postnatal days in preterm newborns with and without patent ductus arteriosus. Balkan Med J. 2014. 31: 230 – 234 | Outcome not clearly described |
| 11 | 124 | Y Katayama, H Minami, M Enomoto, T Takano, S Hayashi and YK Lee. Antenatal magnesium sulfate and the postnatal response of the ductus arteriosus to indomethacin in extremely preterm neonates. Journal of perinatology. 2011. 31: 21 – 24 | All received prophylactic indomethacin |
| 12 | 140 | Hany Aly, Wael Lotfy, Nadia Badrawi, Mohamed Ghawas, Iman Ehsan Abdel-Meguid, and Tarek A. Hammad. Oral ibuprofen and ductus arteriosus in premature infants: a randomized pilot study. Am J Perinatol. 2007. 24: 267 – 270 | Patients where to old according to our eligibility criteria |
| 13 | 165 | Patrick A. Flynn, Ralph L. da Graca, Peter A.M., Mirjana Nesin and Charles S. Kleinman. The use of a bedside assay for plasma B-type natriuretic peptide as a biomarker in the management of patent ductus arteriosus in premature neonates. J pediatr. 2005. 147: 38 – 42 | Outcome data not relevant |
| 14 | 179 | Ana I. Mouzinho, Charles R. Rosenfeld and Richard Risser. Symptomatic patent ductus arteriosus in very-low-birth-weight infants: 1987 – 1989. Early human development. 1991. 27: 65 – 77 | Not clearly when ultrasound was performed |
| 15 | 187 | Scott Tschuppert, Carsten Doell, Romaine Arlettaz-Mieth, Oskar Baenziger, Valentin Rousson, Christian Balmer, René Prêtre, and Ali Dodge-Khatami. The effect of ductal diameter on surgical and medical closure of patent ductus arteriosus in preterm neonates: size matters. Surgery for congenital heart disease. 2008. 135: 78 – 82 | Not clearly described when ultrasound was performed and when ductus was closed |
| 16 | 188 | Selcen Yaroğlu Kazancı, Alper Güzeltaş, Sultan Kavucuoğlu. The relation between brain natriuretic peptide and patent ductus arteriosus in premature infants. Turk Pediatr Ars. 2012. 47: 90 – 94 | Not clearly by who the ductus closed |
| 17 | 203 | Franco Bagnoli, Annalisa Rossetti, Gabriele Messina, Annalisa Mori, Martina Casucci & Barbara Tomasini. Treatment of patent ductus arteriosus (PDA) using ibuprofen: renal side-effects in VLBW and ELBW newborns. The journal of maternal-fetal and neonatal medicine. 2013. 26: 423 – 429 | Closed ductus not described in control group |
| 18 | 216 | Ilene R. S. Sosenko, M. Florencia Fajardo, Nelson Claure, and Eduardo Bancalari. Timing ofpatent ductus arteriosus trtreatmentnd respiratory outcome in premature infants: A double-blind randomized controlled trial. The journal of pediatrics. 2012. 160: 929 – 935 | Not clearly described when ultrasound was performed and when ductus was closed |
| 19 | 218 | Dan Dang, Dongxuan Wang, Chuan Zhang, Wenli Zhou, Qi Zhou, Hui Wu. Comparison of oral paracetamol versus ibuprofen in premature infants with patent ductus arteriosus: A randomized controlled trial. PLoS ONE. 2013. 8: 1 – 5 | Not clearly described when ductus was closed |
| 20 | 227 | L Cordero, CA Nankervis, D DeLooze and PJ Giannone. Indomethacin prophylaxis or expectant treatment of patent ductus arteriosus in extremely low birth weight infants. Journal of perinatology. 2007. 27: 158 – 163 | Expectant group receives also treatment |
| 21 | 228 | M Laughon, C Bose and R Clark. Treatment strategies to prevent or close a patent ductus arteriosus in preterm infants and outcomes. Journal of perinatology. 2007. 27: 164 – 170 | Not clearly when ultrasound was performed |
| 22 | 232 | Shen C.T. Prognostic value of color Doppler echocardiographic findings in premature newborns with patent ductus arteriosus. Acta paediatr sin. 1997. 38: 104 – 110 | Full text not available |
| 23 | 239 | Ekici F, Atasay B, Günlemez A, Naçar N, Tutar E, Atalay S, Eyileten Z, Uysalel A, Arsan S. Management of patent ductus arteriosus in preterm infants. Anadolu Kardiyol Derg. 2006. 6: 28 – 33 | Not clearly when ultrasound was performed |
| 24 | 245 | Nihat Demir, Ibrahim Ece, Erdal Peker, Sultan Kaba, Lokman Ustyol, Ragip Balahoroglu, Oguz Tuncer. Impact of patent ductus arteriosus and subsequent therapy with ibuprofen on the release of S-100B and oxidative stress index in preterm infants. Med Sci Monit. 2014. 20: 2799 – 2805 | Ductus not described as closed, but as hsPDA and no hsPDA |
| 25 | 255 | Hasan Kahveci, Murat Ciftel, Salih Aydemir, Cuneyt Tayman, Osman Yılmaz, Fuat Laloglu, Nazan Kavas, Esra Laloglu, Abdulah Erdil, Hulya Aksoy. Relationship between hemodynamically significant ductus arteriosus and ischemia-modified albumin in premature infants. Ind J Clin Biochem. 2016. 31 (2); 231 – 236 | Ductus not described as closed, but as hsPDA and no hsPDA |
| 26 | 258 | Ahmed Cherif, Sami Jabnoun and Naima Khrouf. Oral ibuprofen in early curative closure of patent ductus arteriosus in very premature infants. Am J perinatal. 2007. 24: 339 – 346 | Not specified how many had spontaneous closure |
| 27 | 266 | DJ O'Rourke, A EL‐Khuffash, C Moody, K Walsh, EJ Molloy. Patent ductus arteriosus evaluation by serial echocardiography in preterm infants. Acta paediatrica. 2008. 97: 574 – 578 | Ductus not described as closed, but on clinical grounds. |
| 28 | 271 | Laura Bourgoin, Cecile Cipierre, Veronique Gournay, Geraldine Gascoin, Quentin Hauet, Jean-Christophe Rozé, Helene Basset, Cyril Flamant. Neurodevelopmental outcome at 2 years of age according to patent ductus arteriosus management in very preterm infants. Neonatology. 2016. 109: 139 – 146 | Ductus not described as closed, but as no hsPDA |
| 29 | 272 | Shahnaz Pourarian, Sirous Cheriki, Deepak Sharma, Farhad Bijanzadeh, Nazanin Farahbakhsh. Prevalence and risk factors associated with the patency of ductus arteriosus in premature neonates: A prospective observational study from Iran. The journal of maternal-fetal and neonatal medicine. 2016. 1 – 5 | Patients where to old according to our eligibility criteria |
| 30 | 273 | Anastasia Varvarigou, Claudette L. Bardin, Kay Beharry, Sylvian Chemtob, Apostolos Papageorgiou, Jacob V. Aranda. Early ibuprofen administration to prevent patent ductus arteriosus in premature newborn infants. JAMA. 1996. 275: 539 – 544 | Ductus not described as closed, but as no hsPDA |
| 31 | 313 | S. Supapannachart, P. Knowsathit, B. Patchakapati. Indomethacin prophylaxis for patent ductus arteriosus (PDA) in infants with a birth weight of less than 1250 grams. J. Med Assoc Thai. 1999. 82: 87 – 92 | Full text not available |
| 32 | 355 | Musoke, RN; Anabwani, GA. Patent ductus arteriosus in African preterm infants. East Afr Med J. 1991. 68: 637 – 641 | Full text not available |
| 33 | 364 | H Holmstrom, C Hall and E Thaulow. Plasma levels of natriuretic peptides and hemodynamic assessment of patent ductus arteriosus in preterm infants. Acta paediatr. 2001. 90: 184 – 191 | Not specified when ductus was exactly closed |
| 34 | 391 | Kluckow M, Evans N. Early echocardiographic prediction of symptomatic patent ductus arteriosus in preterm infants undergoing mechanical ventilation. Journal of pediatrics. 1995. 127: 774 – 779 | Not specified when ductus was exactly closed |
| 35 | 406 | Paola Lago, Sabrina Salvadori, Francesca Opocher, Silvia Ricato, Lino Chiandetti, Anna Chiara Frigo. Continuous infusion of ibuprofen for treatment of patent ductus arteriosus in very low birth weight infants. Neonatology. 2014. 105: 46 – 54 | Ductus not described as closed, but as hsPDA and no hsPDA |
| 36 | 421 | RK Kumar and VYH Yu. Prolonged low-dose indomethacin therapy for patent ductus arteriosus in very low birthweight infants. J. Paediatr. Child Health. 1997. 33: 38 – 41 | Ductus not described as closed, but as hsPDA and no hsPDA |
| 37 | 434 | Se In Sung, Yun Sil Chang, Ji Young Chun, Shin Ae Yoon, Hye Soo Yoo, So Yoon Ahn and Won Soon Park. Mandatory closure versus nonintervention for patent ductus arteriosus in very preterm infants. Journal of pediatrics. 2016. 177: 66 – 71 | Not specified when ductus was exactly closed and ultrasound was performed |
| 38 | 438 | S Ramakrishnan, YM Heung, J Round, TP Morris, P Collinson, AF Williams. Early N-terminal pro-brain natriuretic peptide measurements predict clinically significant ductus arteriosus in preterm infants. Acta paediatrica. 2009. 98: 1254 – 1259 | Not specified how many had spontaneous closure |
| 39 | 489 | Barbara Schmidt, Robin S. Roberts, Avroy Fanaroff, Peter Davis, Haresh M. Kirpalani, Chuks Nwaesei, Michael Vincer and the TIPP investigator. Indomethacin prophylaxis, patent ductus arteriosus and the risk of bronchopulmonary dysplasia: further analyses from the trial of indomethacin prophylaxis in preterms (TIPP). J. Pediatr. 2006. 148: 730 – 734 | Not specified when ductus was exactly closed and ultrasound was performed |
| 40 | 492 | Shahnaz Pourarian, Deepak Sharma, Sirous Cheriki, Farhad Bijanzadeh and Nazanin Farahbakhsh. To evaluate the prevalence of symptomatic and non-symptomatic ductus arteriosus and accuracy of physical signs in diagnosing PDA in preterm infants using blinded comparison of clinical and echocardiographic findings during the first week of life: A prospective observational study from Iran. The journal of maternal-fetal and neonatal medicine. 2016. 1 – 5 | Patients where to old according to our eligibility criteria |
| 41 | 493 | Girija Natarajan, Seetha Shankaran, Scott A. McDonald, Abhik Das, Richard A. Ehrenkranz, Ronald N. Goldberg, Barbara J. Stoll, Jon E. Tyson, Rosemary D. Higgins, Diana Schendel, David M Hougaard, Kristin Skogstrand, Poul Thorsen, Waldemar A. Carlo. Association between blood spot transforming growth factor-B and patent ductus arteriosus in extremely low-birth weight infants. Pediatrc cardiol. 2013. 34: 149 – 154 | Not specified when ultrasound was performed and not described what no PDA is |
| 42 | 494 | Lucia Mirea, Koravangattu Sankaran, Mary Seshia, Arne Ohlsson, Alexander C. Allen, Khalid Aziz, Shoo K. Lee and Prakesh S. Shah on behalf of the Canadian Neonatal Network. Treatment of patent ductus arteriosyus and neonatal mortality/morbidities: adjustment for treatment selection bias. The journal of pediatrics. 2012. 161: 689 – 694 | Not specified when ultrasound was performed |
| 43 | 502 | N. Margreth van der Lugt, Enrico Lopriore, Regina Bökenkamp, Vivianne E. H. J. Smits-Wintjens, Sylke J. Steggerda, Frans J. Walther. Repeated courses of ibuprofen are effective in closure of a patent ductus arteriosus. Eur J Pediatr. 2012. 171: 1673 – 1677 | Not specified when ultrasound was performed |
| 44 | 511 | Bai-Horng Su, Ching-Tien Peng, Chang-Hai Tsai. Echocardiographic flow pattern of patent ductus arteriosus: a guide to indomethacin treatment in premature infants. Arch Dis Child fetal neonatal ed. 1999. 81: F197 – F200 | Almost everyone received treatment |
| 45 | 537 | Afif F. El-Khuffash and Eleanor J. Molloy. Influence of a patent ductus arteriosus on cardiac troponin T levels in preterm infants. J. pediatr. 2008. 153: 350 – 353 | Duplicate |
| 46 | 541 | Mark D. Reller, Michael A Colasurdo, Mary J Rice, Robert W. McDonald. The timing of spontaneous closure of the ductus arteriosus in infants with respiratory distress syndrome. Am J Cardiol. 1990. 66: 75 – 78 | Patients where to old according to our eligibility criteria |
| 47 | 564 | Pei-Chen Tsao, Shu-Jen Chen, Chia-Feng Yang, Yu-Sheng Lee, Mei-Jy Jeng, Wen-Jue Soong, Pi-Chang Lee, Jen-Her Lu, Betau Hwang, Ren-Bin Tang. Comparison of intravenous and enteral indomethacin administration for closure of patent ductus arteriosus in extremely-low-birth-weight infants. J. Chin Med Assoc. 2010. 73: 15 – 20 | Not specified when ultrasound was performed |
| 48 | 626 | M.P. Carboni, R.E. Ringel. Ductus arteriosus in premature infants beyond the second week of life. Pediatr Cardiol. 1997. 18: 372 – 375 | Ultrasound performed to late according to our inclusion criteria |
| 49 | 630 | JW Kaempf, YX Wu, AJ Kaempf, AM Kaempf, L Wang and G Grunkemeier. What happens when the patent ductus arteriosus is treated less aggressively in very low birth weight infants? Journal of perinatology. 2012. 32: 344 – 348 | Not specified when ultrasound was performed |
| 50 | 636 | Istemi Han Celik, Omer Erdeve, Gamze Demırel, Fuat Emre Canpolat, Ugur Dilmen. Elevated urinary NT-proBNP after pharmacological closure of patent ductus arteriosus in very low birth weight infants. Early Human Development. 2013. 89: 187 – 189 | Not specified when ductus was exactly closed |
| 51 | 676 | Gozde Kanmaz, Omer Erdeve, Fuat Emre Canpolat, Serife Suna Oğuz, Nurdan Uras, Nahide Altug, Ben Greijdanus, Uğur Dilmen. Serum ibupforen levels of extremely preterm infants treated prophylactically with oral ibuprofen to prevent patent ductus arteriosus. Eur J Clin Pharmacol. 2013. 69: 1075 – 1081 | Not specified when ductus was exactly closed and not described what no PDA is |
| 52 | 684 | Surmeli-Onay O, Yurdakok M, Karagoz T, Erkekoglu P, Ertugrul I, Takci S, Giray BK, Aykan HH, Korkmaz A, Yigit S. A new approach to an old hypothesis; phototherapy does not affect ductal patency via PGE2 and PGI2. Journal of maternal-fetal and neonatal medicine. 2015. 28: 16 – 22 | Not specified when ultrasound was performed |
| 53 | 706 | Dawn M. Weiss, Jeffrey R. Kaiser, Christopher Swearingen, Sadia Malik and Ritu Sachdeva. Association of antegrade pulmonary artery diastolic velocity with spontaneous closure of the patent ductus arteriosus in extremely low birth weight infants. Am J Perinatol. 2015. 32 (13): 1217 – 1224 | Not specified when ultrasound was performed |
| 54 | 710 | Nick Evans and Parvathi lyer. Assessment of ductus arteriosus shunt in preterm infants supported by mechanical ventilation: Effect of interatrial shunting. J. pediatr. 1994. 125: 778 – 785 | Duplicate |
| 55 | 723 | F. J. Weir, A. Ohlsson, T. L. Myhr, K. Fong, M. L. Ryan. A patent ductus arteriosus is associated with reduced middle cerebral artery blood flow velocity. Eur J Pediatr. 1999. 158: 484 – 487 | Not specified when ultrasound was performed |
| 56 | 728 | Sara B. DeMauro, Meryl S. Cohen, Sarah J. Ratcliffe, Soraya Abbasi and Barbara Schmidt. Serial echocardiography in very preterm infants: A pilot randomized trial. Acta paediatr. 2013. 102 (11): 1048 – 1053 | Prophylactic treatment was taken in account |
| 57 | 729 | Abdollah Jannatdoust, Mahmoud Samadi, Saadollah Yeganehdoust, Mohammad Heydarzadeh, Hossein Alikhah, Reza Piri, Mohammad Naghavi-Behzad. Effects of intravenous indomethacin on reduction of symptomatic patent ductus arteriosus cases and decreasing the need for prolonged mechanical ventilation. J Cardiovasc Thorac Res. 2016. 6 (4): 257 – 259 | No placebo group and not specified when ductus closed |
| 58 | 753 | Sophie Vanhaesebrouck, Inge Zonnenberg, Piet Vandervoort, Els Bruneel, Marie-Rose Van Hoestenberghe, Claire Theyskens. Conservative treatment for patent ductus arteriosus in the preterm. Arch Dis Child Fetal Neonatal Ed. 2007. 92: F244 – F247 | Combined group of closed and asymptomatic PDA |
| 59 | 754 | Carlo Dani, Giovanna Bertini, Iuri Corsini, Serena Elia, Venturella Vangi, Simone Pratesi, Firmino F. Rubaltelli. The fate of ductus arteriosus in infants at 23 – 27 weeks of gestation: from spontaneous closure to ibuprofen resistance. Acta paediatrica. 2008. 97: 1176 – 1180 | Not specified when ductus was exactly closed |
| 60 | 763 | S. Alagarsamy, M. Chhabra, M. Gudavalli, A.M. Nadroo, V.G. Sutija, D. Yugrakh. Comparison of clinical criteria with echocardiographic findings in diagnosing PDA in preterm infants. J. Perinat Med. 2005. 33: 161 – 164 | Full text not available |
| 61 | 767 | María Carmen Bravo, Fernando Cabañas, Joan Riera, Elia Pérez-Fernández, José Quero, Jesús Pérez-Rodríguez & Adelina Pellicer. Randomised controlled clinical trial of standard versus echocardiographically guided ibuprofen treatment for patent ductus arteriosus in preterm infants: a pilot study. The journal of maternal-fetal and neonatal medicine. 2014. 27: 904 – 909 | PDA not described as closed but as smaller than 1,5 mm |
| 62 | 769 | C Dani, V Vangi, G Bertini, S Pratesi, I Lori, F Favelli, R Ciuti, A Bandinelli, C Martano, P Murru, H Messner, F Schena and F Mosca. High-dose ibuprofen for patent ductus arteriosus in extremely preterm infants: a randomized controlled study. Clin Pharmacol Ther. 2012. 91: 590 – 596 | Ductus not described as closed, but as no hsPDA |
| 63 | 774 | Laurent Tauzin, Corinne Joubert, Anne-Claire Noel, Antoine Bouissou, Marie-Eve Moulies. Effect of persistent patent ductus arteriosus on mortality and morbidity in very low-birthweight infants. Acta paediatrica. 2012. 101: 419 – 423 | Ductus not described as closed, but as without persistent significant PDA |
| 64 | 775 | V. Zanardo, O. Milanesi, D. Trevisanuto, M. Rizzo, M. Ronconi, G. Stellin, F. Cantarutti. Early screening and treatment of ‘silent’ patent ductus arteriosus in prematures with RDS. J Perinat Med. 1991. 19: 291 – 295 | Full text not available |
| 65 | 788 | W.K. Chan, K.I. Tsou, P.N. Tsao, H.C. Chou, C.H. Wu, W.S. Hsieh. Prophylactic indomethacin in extremely-low-birth-weight infants. Clin Neonatol. 2003. 10: 22 – 26 | Full text not available |
| 66 | 806 | V.Y. Chock, L.A. Rose, J.V. Mante, R. Punn. Near-infrared spectroscopy for detection of a significant patent ductus arteriosus. Pediatr Res. 2016. 80: 675 – 680 | Full text not available |
| 67 | 822 | FJ Weir, A Smith, P Littleton, N Carter and PA Hamilton. Atrial natriuretic peptide in the diagnosis of patent ductus arteriosus. Acta Paediatr. 1992. 81: 672 – 675 | Not specified when ultrasound was performed |
| 68 | 860 | Emily Cohen, Laura Dix, Willem Baerts, Thomas Alderliesten, Petra Lemmers, Frank van Bel. Reduction in cerebral oxygenation due to patent ductus arteriosus is pronounced in small-for-gestational-age neonates. Neonatology. 2017. 111: 126 – 132 | Ductus not described as closed, but as hsPDA and no hsPDA |
| 69 | 861 | Sindhu Sivanandan, Varun Bali, Amuchou Singh Soraisham, Andrei Harabor, Majeeda Kamaluddeen. Effectiveness and safety of indomethacin versus ibuprofen for the treatment of patent ductus arteriosys in preterm infants. Am J Perinatol. 2013. 30: 745 – 750 | Not specified when ultrasound was made and when the ductus was exactly closed |
| 70 | 874 | Ozgur Olukman, Sebnem Calkavur, Gulten Ercan, Fusun Atlihan, Taliha Oner, Vedide Tavli and Nilgun Kultursay. Comparison of oral and intraveneous ibuprofen for medical closure of patent ductus arteriosus: Which one is better? Congenit Heart Dis. 2012. 7: 534 – 543 | Not specified when ductus was exactly closed |
| 71 | 880 | Gilles Cambonie, Anne-Marie Dupuy, Clémentine Combes, Marie Vincenti, Renaud Mesnage, Jean-Paul Cristol. Can a clinical decision rule help ductus arteriosus management in preterm neonates? Acta paediatrica. 2012. 101: 213 – 218 | Not specified when ductus was exactly closed |
| 72 | 881 | H. Popat, V. Kapoor and J. Travadi. Patent ductus arteriosus in infants < 29 weeks gestation – outcomes and factors affecting closure. Indian Pediatr. 2012. 49: 615 – 620 | Ductus not only described as closed, but also as no sPDA |
| 73 | 887 | Magalie Vidal, Felicie Ferragu, Sabine Durand, Julien Baleine, Aline Rideau Batista-Novais, Gilles Cambonie. Perfusion index and its dynamic changes in preterm neonates with patent ductus arteriosus. Acta Paediatrica. 2013. 102: 373 – 378 | Not specified how many PDA closed in which patients |
| 74 | 903 | Chitra Ravishankar, Suhas Nafday, Robert S Green, Steven Kamenir, Richard Lorber, Maria Stacewicz-Sapuntzakis, Nancy D. Bridges, Ian R. Holzman and Bruce D. Gelb. A trial of vitamin D therapy to facilitate ductal closure in premature infants. J. Pediatr. 2003. 143: 644 – 648 | Ductus not only described as closed, but also as small PDA |
| 75 | 909 | V. Sangtawesin, C. Sangtawesin, C. Raksasinborisut, K. Sathirakul, W. Kanjanapattanakul, M. Khorana, S. Horpaopan. Oral ibuprofen prophylaxis for symptomatic patent ductus arteriosus of prematurity. J Med Assoc Thailand. 2006. 89: 314 – 321 | Full text not available |
| 76 | 918 | Kalyan Chakravarthy Balla, Vinu John, Suman Rao PN and Kiron Varghese. Perfusion index – bedside diagnosis of hemodynamically significant patent ductus arteriosus. Journal of tropical pediatrics. 2016. 62: 263 – 268 | Ductus not described as closed, but as hsPDA and no hsPDA |
| 77 | 919 | N Nimeri, H Salama. Short-term outcome of different treatment modalities of patent ductus arteriosus in preterm infants. Five years experiences in Qatar. The internet journal of cardiovascular research. 2010. 7 | Not specified when ductus was exactly closed |
| 78 | 928 | I Farombi-Oghuvbu, T Matthews, P D Mayne, H Guerin, J D Corcoran. N-terminal pro-B-type natriuretic peptide: a measure of significant patent ductus arteriosus. Arch Dis Child Fetal neonatal Ed. 2008. 93: 257 – 260 | Not specified when ductus was exactly closed |
| 79 | 953 | Lilian S.R. Sadecka, Cléa R. Leone, Renato S. Procianoy, Ruth Guinsburg, Sergio T.M. Marba, Francisco E. Martinez, Ligia M.S.S. Rugolo, M. Elisabeth L. Moreira, Renato M. Fiori, Ligia L. Ferrari, Jucille A. Menezes, Paulyne S. Venzon, Vânia Q.S. Abdallah, José Luiz M.B. Duarte, Marynea V. Nunes, Leni M. Anchieta, Navantino Alves Filho. Effects of therapeutic approach on the neonatal evolution of very low birth weight infants with patent ductus arteriosus. Jornal de Pediatria. 2014. 90: 616 – 623 | Not clearly when ultrasound was performed |
| 80 | 966 | Outi Tammela, Riitta Ojala, Tiina Iivainen, Visa Lautamatti, Marja-Leena Pokela, Martti Janas, Maila Koivisto and Sami Ikonen. Short versus prolonged indomethacin therapy for patent ductus arteriosus in preterm infants. J. pediatr. 1999. 134: 552 – 557 | Not specified when ductus was exactly closed |
| 81 | 971 | Roberson DA, Silverman NH. Color Doppler flow mapping of the patent ductus arteriosus in very low birthweight neonates: echocardiographic and clinical findings. Pediatr Cardiol. 1994. 15: 219 – 224 | Not specified when ductus was exactly closed |
| 82 | 980 | Ying-Yao Chen, Hsiao-Ping Wang, Jenn-Tzong Chang, Yee-Hsuan Chiou, Yung-Feng Huang, Kai-Sheng Hsieh and Taiwan Premature Infant Development Collaborative Study Group. Perinatal factors in patent ductus arteriosus in very low-birthweight infants. Pediatrics international. 2014. 56: 72 – 76 | Not clearly when ultrasound was performed |
| 83 | 1003 | K Herrman, C Bose, K Lewis, M Laughon. Spontaneous closure of the patent ductus arteriosus in very low birth weight infants following discharge from the neonatal unit. Arch Dis Child Fetal Neonatal Ed. 2009. 94: 48 – 50 | Outcome/spontaneous closure occurred too late |
| 84 | 1010 | Ahmed Cherif, Naima Khrouf, Sami Jabnoun, Chahnez Mokrani, Moez Ben Amara, Nedia Guellouze, Samia Kacem. Randomized pilot study comparing oral ibuprofen with intravenous ibuprofen in very low birth weight infants with patent ductus arteriosus. Pediatrics. 2008. 122: 1256 – 1261 | Not clearly when ultrasound was performed |
| 85 | 1022 | Majeda S. Hammoud, Hassan A. Elsori, Emad-Aldeen M. Hanafi, Adel A. Shalabi, Ibrahim A. Fouda, Lakshmi V. Devarajan. Incidence and risk factors associated with the patency of ductus arteriosus in preterm infants with respiratory distress syndrome in Kuwait. Saudi Med J. 2003. 24: 982 – 985 | Ductus not described as closed, but as significant PDA and no significant PDA |
| 86 | 1024 | Nidhi A. Shah, Nancy K. Hills, Nahid Waleh, Donald McCurnin, Steven Seidner, Sylvain Chemtob and Ronald Clyman. Relationship between circulating platelet counts and ductus arteriosus patency after indomethacin treatment. The journal of pediatrics. 2011. 158: 919 – 923 | All received prophylactic indomethacin |
| 87 | 1044 | Outi Aikio, Pia Härkin, Timo Saarela & Mikko Hallman. Early paracetamol treatment associated with lowered risk of persistent ductus arteriosus in very preterm infants. The journal of maternal-fetal and neonatal medicine. 2014. 27: 1252 – 1256 | Not specified when ductus was exactly closed |
| 88 | 1052 | Maria Pilar Bas-Suárez, Gema Esther González-Luis, Pedro Saavedra, Eduardo Villamor. Platelet counts in the first seven days of life and patent ductus arteriosus in preterm very low-birth-weight infants. Neonatology. 2014. 106: 188 – 194 | Ductus not described as closed, but as hsPDA and no hsPDA |
| 89 | 1063 | J M Brooks, J N Travadi, S K Patole, D A Doherty, K Simmer. Is surgical ligation of patent ductus arteriosus necessary? The Western Australian experience of conservative management. Arch Dis Child Fetal Neonatal Ed. 2005. 90: F235 – F239 | Not clearly when ultrasound was performed |
| 90 | 1074 | Joon Sik Kim and Eun Jung Shim. B-type natriuretic peptide assay for the diagnosis and prognosis of patent ductus arteriosus in preterm infants. Korean circulation journal. 2012. 42: 192 – 196 | Outcome not relevant: hsPDA not definitively closed |
| 91 | 1080 | Shehab Ahmed Alenazi. N-terminal pro-brain natriuretic peptide measurements in hemodynamically significant patent ductus arteriosus in preterm infants. Pak J Med Sci. 2016. 32: 580 – 584 | Ductus not described as closed, but as hsPDA and no hsPDA |
| 92 | 1104 | Przemko Kwinta, Andrzej Rudziński, Piotr Kruczek, Zbigniew Kordon,  Jacek Jόzef Pietrzyk. Can early echocardiographic findings predict patent ductus arteriosus. Neonatology. 2009. 95: 141 – 148 | Ductus not described as closed, but as hsPDA and no hsPDA |
| 93 | 1108 | Nick Evans, Parvathi Iyer. Longitudinal changes in the diameter of the ductus arteriosus in ventilated preterm infants: correlation with respiratory outcomes. Archives of disease in childhood. 1995. 72: F156 – F161 | Duplicate |
| 94 | 1117 | Boo N Y, Mohd-Amin I, Bilkis A A, Yong-Junina F. Predictors of failred closure of patent ductus arteriosus with indomethacin. Singapore Med J. 2006. 47: 763 – 768 | Not clearly when ultrasound was performed |
| 95 | 1131 | Cathy Hammerman, Joram Glaser, Michael Kaplan, Michael S. Schimmel, Benjamin Ferber and Arthur I. Eidelman. Indomethacin tocolysis increases postnatal patent ductus arteriosus severity. Pediatrics. 1998. 102: 1 – 4 | Not clearly when ultrasound was performed and ductus closed |
| 96 | 1171 | Kathryn Browning Carmo, Nick Evans and Mary Paradisis. Duration of indomethacin treatment of the preterm patent ductus arteriosus as directed by echocardiography. The journal of pediatrics. 2009. 155: 819 – 822 | Ductus not described as only closed, but also not significant PDA |
| 97 | 1213 | T.H. Lai, W.J. Soong, B. Hwang. Indomethacin for the prevention of symptomatic patent ductus arteriosus in very low birth weight infants. Zhonghua Min Guo Xiao Er Ke Yi Xue Hui Za Zhi. 1990. 31: 17 – 23 | Full text not available |
| 98 | 1274 | F. Bagnoli, A. Rossetti, M. Casucci, A. Mori. Aminoterminal B-type natriuretic peptide (NT-proBNP) in the therapy of patent ductus arteriosus. Minerva Pediatr. 2010. 62: 67 – 70 | Full text not available |
| 99 | 1513 | Martin Kluckow and Nick Evans. Ductal shunting, high pulmonary blood flow, and pulmonary hemorrhage. J. Pediatr. 2000. 137: 68 – 72 | Not clearly when ultrasound was performed and ductus closed |
| 100 | 1520 | P.P. Thankavel, C.R. Rosenfeld, L. Christie, C. Ramaciotti. Early echocardiographic prediction of ductal closure in neonates <30 weeks gestation. J. perinatal. 2013. 33: 45 – 51 | Ductus not only described as closed, but also as small PDA |
| 101 | 1530 | YoungAh Youn, Ju-Young Lee, Jung Hyun Lee, So-Young Kim, In Kyung Sung, Jae Young Lee. Impact of patient selection on outcomes of PDA in very low birth weight infants. Early human development. 2013. 89: 175 – 179 | Not clearly when ultrasound was performed and ductus closed |
| 102 | 1539 | M El Hajjar, G Vaksmann, T Rakza, G Kongolo, L Storme. Severity of the ductal shunt: a comparison of different markers. Arch Dis Child Fetal Neonatal Ed. 2005. 90: F419 – F422 | Not clearly when ultrasound was performed |
| 103 | 1600 | P. Arun Kumar Nair, Mangalore Govind Pai, Hesham Abdel Rahim Gazal, David Eustace Da Costa and Saleh Mohammed Al Khusaiby. Indomethacin prophylaxis for intraventricular hemorrhage in very low birth weight infants. Indian pediatr. 2004. 41: 551 – 558 | Not clearly when ultrasound was performed |
| 104 | 1740 | Ted Trus, Andrea L. Winthrop, Steven Pipe, Jay Shah, Jacob C. Langer and George Y.P. Lau. Optimal management of patent ductus arteriosus in the neonate weighing less than 800 gram. J pediatr surg. 1993. 28: 1137 – 1139 | Not clearly when ultrasound was performed |
| 105 | 1745 | Robert J. Couser, Ronald E. Hoekstra, T. Bruce Ferrara, Gregory B. Wright, Allison K. Cabalka, John E. Connett. Neurodevelopmental follow-up at 36 months’ corrected age of preterm infants treated with prophylactic indomethacin | Not clearly when ultrasound was performed and ductus closed |
| 106 | 1747 | Justin Richards, Alice Johnson, Grenville Fox and Morag Campbell. A second course of ibuprofen is effective in the closure of a clinically significant PDA in ELBW infants. Pediatrics. 2009. 124: e287 – e293 | Ductus not described as closed, but as hsPDA and no hsPDA |
| 107 | 1793 | Martin Kluckow, Michele Jeffery, Andy Gill, Nick Evans. A randomized placebo-controlled trial of early treatment of the patent ductus arteriosus. Arch dis child fetal neonatal ed. 2014. 99: F99 – F104 | Ductus not described as only closed, but also small PDA |
| 108 | 1874 | Pees C, Walch E, Obladen M, Koehne P. Echocardiography predicts closure of patent ductus arteriosus in response to ibuprofen in infants less than 28 week gestational age. Early Hum Dev. 2010. 86: 503 – 508 | All patients received treatment |
| 109 | 1934 | S Chen, T Tacy and R Clyman. How useful are B-type natriuretic peptide measurements for monitoring changes in patent ductus arteriosus shunt magnitude? Journal of perinatology. 2010. 30: 780 – 785 | Not clearly when ultrasound was performed |
| 110 | 2020 | Barbara Schmidt, Mary Seshia, Seetha Shankaran, Lindsay Mildenhall, Jon Tyson, Kei Lui, Tai Fok and Robin Roberts for the Trial of Indomethacin Prophylaxis in Preterms Investigators. Effects of prophylactic indomethacin in extremely low birth weight infants with and without adequate exposure to antenatal steroids. Arch Pediatr Adolesc Med. 2011. 165: 642 – 646 | Not clearly when ultrasound was performed |
| 111 | 2068 | A.V. Rivera Sepulveda, N. Claudio, L. Garcia-Fragoso. Closure of patent ductus arteriosus in low-birth weight infants based on three management strategies. Bol Asoc Med P R. 2013. 105: 14 – 19 | Full text not available |
| 112 | 2236 | A.F. El-Khuffash, M. Amoruso, M. Culliton, E.J. Molloy. N-terminal pro-B-type natriuretic peptide as a marker of ductal haemodynamic significance in preterm infants: A prospective observational study. Arch dis child fetal neonatal ed. 2007. 92: F421 – F422 | Ductus not described as only closed |
| 113 | 2598 | Gianluca Lista, Silvia Bianchi, Savina Mannarino, Federico Schena, Francesca Castoldi, Mauro Stronati, Fabio Mosca. Velocity time integral for right upper pulmonary vein in VLBW infants with patent ductus arteriosus. Clinics. 2016. 71: 580 – 585 | Ductus not described as closed, but as hsPDA and no hsPDA |
| 114 | 2741 | Karl Wilhelm Olsson, Anders Jonzon, and Richard Sindelar. A high ductal flow velocity is associated with successful pharmacological closure of patent ductus arteriosus in infants 22 – 27 weeks gestational age. Crit Care Res Pract. 2012. | No placebo of spontaneous closure. |
| 115 | 2794 | Jin A Lee, Myo-Jing Kim, Sohee Oh and Byung Min Choi. Current status of therapeutic strategies for patent ductus arteriosus in very-low-birth-weight infants in Korea. J Korean Med Sci. 2015. 30: S59 – S66 | Not clearly when ductus was closed |
| 116 | 4034 | D A Osborn, N Evans, M Kluckow. Effect of early targeted indomethacin on the ductus arteriosus and blood flow to the upper body and brain in the preterm infant. Arch Dis Child Fetal Neonatal Ed. 2003. 88: F477 – F482 | Cross over trial and al treated with indomethacin |
| 117 | 4285 | C.S. Lee, B. Hwang, J.H. Lu, W.J. Soong, S.J. Chen. Symptomatic patent ductus arteriosus in very low birth weight infants. Chung Hua I Hsueh Ysa Chih. 1998. 61: 93 – 98 | Full text not available |
| 118 | 5033 | Knight, Alkindi, Buksh, Kuschel C, Skinner, J. Placebo-controlled pilot trial of indomethacin in preterm infants with a patent ductus arteriosus. Journal of paediatrics and child health. 2011. 47: 88 | Congres text |
| 119 | 5105 | Lin Xin-Zhu, Chen Han-Qiang, Zheng Zhi, Li Ya-Dan, Lai Ji-Dong, Huang Li-Han. Therapeutic effect of early administration of oral ibuprofen in very low birth weight infants with patent ductus arteriosus. Zhongguo dang dai er ke za zhi. 2012. 14: 502 – 505 | Article not in English |
| 120 | 5166 | M. Kluckow, N. Evans, A. Gill, M. Jeffery. Ductal echocardiographic targeting and early closure trial (DETECT): A pilot randomised controlled trial. Journal of paediatrics and child health. 2012. 48: 43 – 44 | Oral abstract |
| 121 | 6379 | Sheng-Ling Jan, Betau Hwang, Yun-Ching Fu & Ching-Shiang Chi. Prediction of ductus arteriosus closure by neonatal screening echocardiography. The internation journal of cardiovascular imaging. 2004. 20: 255 – 262 | Patients where to old according to our eligibility criteria |
| 122 | 7743 | Ozmert M. A. Ozdemir, Mustafa Dogan, Kazım Kucuktascı, Hacer Ergin, Ozlem Sahin. Paracetamol threrapy for patent ductus arteriosus in premature infants: a chance before surgical ligation. Pediatr Cardiol. 2014. 35: 376 – 279 | Not clearly when ultrasound was performed and case series report |
| 123 | 7773 | Matthew C. Schwartz, David Nykanen, Lawrence H. Winner, Jose Perez, Michael McMahan, Hamish M. Munro and Sukumar Suguna Narasimhulu. Transcatheter patent ductus arteriosus occlusion in small infants. Congenit heart Dis. 2016. 11: 647 – 655 | Patients were too late included in the stude and not clearly when ultrasound was performed |
| 124 | 7777 | Ines Tofe Valera, Marıa Pilar Jaraba Caballero, Marıa Dolores Ruiz Gonzalez, Marıa Victoria Rodrıguez Benıtez and Marıa Jose Parraga Quiles. The role of paracetamol for closing patent ductus arteriosus. A challenging alternative for ductal closure? | Case report |
| 125 | 7778 | John N. van den Anker, Karel Allegaert. Acetaminphen to prevent symptomatic patent ductus arteriosus: Another drug bites the dust? The journal of pediatrics. 2016. 177: 7 – 9 | Descriptive study about medication |
| 126 | 7781 | Adis Medical Writers. Consider pharmacological treatment to close patent ductus arteriosus in preterm infatns when the condition is haematologically significant. Drugs Ther Perspect. 2017. 33: 22 – 25 | Descriptive study about medication |
| 127 | 7783 | Rizky Adriansyah, Nikmah S. Idris, Mulyadi M. Djer, Sukman T. Putra, Rinawati Rohsiswatmo. Intravenous paracetamol and patent ductus arteriosus in closure in preterm infants. Paediatrica Indonesiana. 2017. 57: 198 – 203 | Not clearly when ultrasound was performed |
| 128 | 7788 | Romaine Arlettaz. Echocardiographic evaluation of patent ductus arteriosus in preterm infants. Frontiers in pediatrics. 2017. 5: 147 | Echocardiographic review |
| 129 | 7789 | Alejandro Avila-Alvarez, Marta Serantes Lourido, Rebeca Barriga Bujan, Carolina Blanco Rodriguez, Francisco Portela-Torron, Victor Bautista-Hernandez. Surgical closure of patent ductus arteriosus in premature neonates: does the surgical technique affect the outcome? Anales de Pediatria. 2017. 86: 277 – 283 | Outcome not described and not clearly when ultrasound was performed |
| 130 | 7791 | Eduardo Bancalari, Deepak Jain. Management of patent ductus arteriosus: Are we looking at the right outcomes? Journal of pediatrics. 2017. 182: 10 – 11 | Not a trial |
| 131 | 7794 | Payman Barikbin, Hannes Sallmon, Silke Wilitzki, Joachim Photiadis, Christoph Bührer, Petra Koehne and Gerd Schmalisch. Lung function in very low birth weight infants after pharmacological and surgical treatment of patent ductus arteriosus – a retrospective analysis. BMC pediatrics. 2017. 17: 5 | All infants received treatment |
| 132 | 7797 | William E. Benitz. Hey, Doctor, Leave the PDA alone. Pediatrics. 2017. 140: | Commentary |
| 133 | 7802 | Nansi S. Boghossian, Barbara T. Do, Edward F. Bell, John M. Dagle, Jane E. Brumbaugh, Barbara J. Stoll, Betty R. Vohr, Abhik Das, Seetha Shankaran, Pablo J. Sanchez, Myra H. Wyckoff, M. Bethany Ball, for the Eunice Kennedy Shriver National Institute of Child Health and Human Development Neonatal Research Network. Efficacy of pharmacologic closure of patent ductus arteriosus in small-for-gestational-age extremely preterm infants. Early human development. 2017. 113: 10 – 17 | Not clearly when ultrasound was performed |
| 134 | 7805 | Colm R. Breatnach, Afif El-Khuffash, Adam James, Naomi McCallion, Orla Franklin. Serial measures of cardiac performance using tissue Doppler imaging verlocity in preterm infants <29 weeks gestations. Early human development. 2017. 108: 33 – 39 | Outcome not described as closed but as no hsPDA |
| 135 | 7815 | Clarissa de Albuquerque Botura, Bruno Ambrosio da Rocha, Thiely Balensiefer, Franciele Queiroz Ames, Ciomar Aparecida Bersani-Amado, Roberto Kenji Nakamura Cuman. Oral pharmacological treatment for patent ductus arteriosus in premature neonates with hemodynamic repercussions. Asian Pacific Journal of tropical medicine. 2017. 10: 1080 – 1083 | Case report serie |
| 136 | 7816 | Koert de Waal, Nilkant Phad, Nick Collins, Andrew Boyle. Congenital heart disease. 2017. 12: 364 – 372 | Outcome not described as closed but as no hsPDA |
| 137 | 7827 | Abd El-Rahman El-Mashad, Heba El-Mahdy, Doaa El Amrousy, Marwa Elgendy. Comparative study of the efficacy and safety of paracetamol, ibuprofen and indomethacin in closure of patent ductus arteriosus in preterm neonates. Eur J Pediatr. 2017. 176: 233 – 240 | All patients received medication |
| 138 | 7837 | Elizabeth E. Foglia, Barbara Schmidt. Neurodevelopmental outcomes following surgical ligation for patent ductus arteriosus among extremely preterm infants case closed? JAMA pediatrics. 2017. 171: 422 – 424 | Opinion, editorial |
| 139 | 7838 | Silvia Foligno, Paola Giliberti, Francesca Landolfo, Veronica Pannone, Pietro Bagolan, Andrea Dotta and Guglielmo Salvatori. Monitoring near-infrared spectroscopy during treatment with ibuprofen to predict splanchnic ischemia in a newborn with patent ductus arteriosus. Iran J Pediatr. 2017. 27: e8806 | Case report |
| 140 | 7840 | Franco Gálvez-Cancino. Paracetamol (acemaniofeno) intravenoso para cierre de conducto arterioso permeable en premturos < 32 semanas de gestacion. Gaceta medica de mexico. 2017. 153: 752 – 756 | Article not in English |
| 141 | 7848 | V. Gournay. Management of patent ductus arteriosus in preterm infants: An evidence-based approach. Arch Pediatr. 2017. 24: 175 – 179 | Article not in English |
| 142 | 7854 | Kai-Hsiang Hsu, Tai-Wei Wu, I-Hsyuan Wu, Mei-Yin Lai, Shih-Yun Hsu, Hsiao-Wen Huang, Tze-Yee Mok, Reyin Lien. Electrical cardiometry to monitor cardiac output in preterm infants with patent ductus arteriosus: a comparison with echocardiography. Neonataology. 2017. 112: 231 – 237 | Outcome not described as closed but as no hsPDA |
| 143 | 7862 | Erik A. Jensen, Martin Keszler, Kevin C. Dysart, Michele C. Walsh, Marie G. Gantz, Benjamin Carper, Matthew M. Laughon, Brenda B. Poindexter, and Barbara Schmidt, Rosemary D. Higgins, Barbara J. Stoll on behalf of the Eunice Kennedy Shriver National Institute of Child Health and Human Development Neonatal Research Network. Association between use of prophylactic indomethacin and the risk for bronchopulmonary dysplasia in extremely preterm infants. Journal of pediatrics. 2017. 186: 34 – 40 | Not clearly when ultrasound was performed and nothing reported on ductal closure |
| 144 | 7863 | Antonio Jimenez-Aceituna, Jose M. Arribas-Leal, Joaquin Perez-Andreu y Sergio J. Canovas-Lopez. Escala de riesgo Artisoteles como factor predictor de mortalidad en el cierre quirurgico de persistencia de ductus arterioso en permaturos. Circ Cardiov. 2017. 24: 222 – 227 | Article not in English |
| 145 | 7866 | Soo Jung Kang, Young Sun Cho, Seo Jung Hwang, Hyo Jin Kim. Outcomes of left ventricular function according to treatment response for a patent ductus arteriosus in preterm infants. J. Cardiovasc Ultrasound. 2017. 25: 131 – 137 | PDA not described as closed but as no hsPDA |
| 146 | 7871 | Annemarie Kindler, Barbara Seipolt, Antje Heilmann, Ursula Range, Mario Rüdiger and Sigrun Ruth Hofmann. Development of a diagnostic clinical score for hemodynamically significant ductus arteriosus. Frontiers in pediatrics. 2017. 5: 280 | PDA not described as closed but as no hsPDA |
| 147 | 7874 | Ashutosh Kumar, Venkataseshan Sundaram, Rahul Yadav, Tejo Pratap Oleti, Srinivas Murki, Arun Krishna, Mangalabharathi Sundaram, Shiv Sajan Saini, Sourabh Dutta. Oral paracetamol versus oral ibuprofen for closure of haemodynamically significant patent ductus arteriosus in preterm neonates (<32 weeks): a blinded, randomized, active-controlled, non-inferiority trial. BMJ Paediatrics open. 2017. 1 | RCT protocol |
| 148 | 7882 | J.B. Letshwiti, J. Semberova, K. Pichova, E.M. Dempsey, O.M. Franklin, J. Miletin. A conservative treatment of patent ductus arteriosus in very low birth weight infants. Early human development. 2017. 104: 45 – 49 | Article did not met inclusion criteria |
| 149 | 7883 | Philip T. Levy, Afif El-Khuffash. Pulmonary arterial hypertension after ibuprofen treatment in the first week of life? J Pediatr. 2017. 182: 408 – 409 | Letter to the editor |
| 150 | 7887 | Caitlyn M. Luecke, Caren J. Liviskie, Brandy N. Zeller, Zachary A. Vesoulis and Christopher McPherson. Acetaminophen for patent ductus arteriosus in extremely low-birth-weight neonates. J. pediatr pharmacol ther. 2017. 22: 461 – 466 | All patients received medication (acetaminophen) |
| 151 | 7890 | Doff B. McElhinney. Small and preterm infants: the shrinking frontier of thrascatheter patent ductus arteriosus closure. Catheterization and cardiovascular interventions. 2017. 89: 1066 – 1068 | Comments |
| 152 | 7892 | C. Michel Macíasa, S. Carrera Muinos, L.A. Fernández Carrocera, O. Guido Ramíres, A. Machuca Vaca, G. Cordero González. Evolucion de los recien nacidos prematuros con cierre quirurgico del conducto arterioso. Perinatologia y reproduccion humana. 2017. 31: 113 – 118 | Article not in English |
| 153 | 7898 | Patrice Morville and Ahmad Akhavi. Transcatheter closure of hemodynamic significant patent ductus arteriosus in 32 premature infants by ductal occluder additional size-ADOIIAS. Catheterizaion and cardiovascular interventions. 2017. 90: 612 – 617 | All patients were treated |
| 154 | 7901 | Nazmi Narin, Özge Pamukçu, Ali Baykan, Mustafa Argun, Abdullah Özyurt, Adnan Bayram, Kazım Üzüm. Transcatheter closure of PDA in premature babies less than 2 kg. Anatol J Cardiol. 2017. 17: 147 – 153 | Only patients included who were treated |
| 155 | 7907 | Samantha Ngo, Jochen Profit, Jeffrey B. Gould, Henry C. Lee. Trends in patent ductus arteriosus diagnosis and management for very low birth weight infants. Pediatrics. 2017. 139 | Not clearly when ultrasound was performed and ductus closed |
| 156 | 7910 | Shahab Noori and S. Ram Kumar. Pre-dicting post-ligation syndrome. J. Thorac cardiovasc surg. 2017. 154: 2060 – 2061 | Editorial commentary |
| 157 | 7921 | Sarah J. Ratcliffe, Laurie G. Sherlock, Clyde J. Wright. Oral paracetamol or oral ibuprofen to close the ductus arteriosus: both ‘work’, but do we know when to use them? Acta paediatr Int J Paediatr. 2017. 106: 1539 | Commentary |
| 158 | 7925 | P. Sadeghi-Moghaddam, M.H. Arjmandnia, A. Heidari, S.M. Mohagheghi-Kamal, M. Aghaali. Comparison of therapeutic effects and side effects of oral ibuprofen and indomethacin on the closure of patent ductus arteriosus in premature infants. J Babol Univ Med Sci. 2017. 19: 7 – 12 | Article not in English |
| 159 | 7926 | İrfan Oğuz Şahin, Canan Yolcu, Ayşegül Elbir Şahin, Mustafa Kara, Yaşar Demirelli, Haşim Olgun, Naci Ceviz. Which criteria are more valuable in defining hemodynamic significance of patent ductus arteriosus in premature infants? Respiratory or echocardiographic? Med Bull Haseki. 2017. 55: 32 – 36 | Not specified when ultrasound was performed and ductus not described as closed, but as hsPDA and hiPDA |
| 160 | 7928 | Rodrigo Salas, Pablo Lavín, Yohanna Rincón, Juan Miranda, María López. Digestive and renal complications in premature infants with patent ductus arteriosus treated with indomethacin and ibuprofen. Rev Chil Pediatr. 2017. 88: 243 – 251 | Not specified when ultrasound was performed and ductus not described as closed |
| 161 | 7932 | Jana Samberova, Jan Sirc, Jan Miletin, Jachym Kucera, Ivan Berka, Sylva Sebkova, Sinead O’Sullivan, Orla Franklin, Zbynek Stranak. Spontaneous closure of patent ductus arteriosus in infants < 1500 g. Pediatrics. 2017; 140 (2): e20164258 | Not specified when ultrasound was performed |
| 162 | 7934 | Jeonghee Shin, Eun Hee Lee, Jee Hyun Lee, Byung Min Choi, Young Sook Hong. Individualized ibuprofen treatment using serial B-type natriuretic peptide measurement for symptomatic patent ductus arteriosus in very preterm infants. Korean J Pediatr. 2017. 60: 175 – 180 | All patients received treatment |
| 163 | 7937 | Jonathan L. Slaughter, Mark A. Klebanoff. Neurodevelopmental outcome in relation to treatment of patent ductus arteriosus. JAMA pediatr. 2017. 171: 1017 – 1018 | Letter to the editor |
| 164 | 7938 | Jonathan L. Slaughter, Patricia B. Reagan, Thomas B. Newman, Mark A. Klebanoff. Comparative effectiveness of nonsteroidal anti-inflammatory drug treatment vs no treatment for patent ductus arteriosus in preterm infants. JAMA pediatrics. 2017. 171. | Not specified when ultrasound was performed and ductus not described as closed |
| 165 | 7939 | Caitlin J. Smith, Kelli K. Ryckman, Timothy M. Bahr and John M. Dagle. Polymorfphisms in CYP2C9 are associated with response to indomethacin among neonates with patent ductus arteriosus. Pediatr Res. 2017. 82: 776 – 780 | All patients received treatment |
| 166 | 7941 | Tomasz Stankowski, Sleiman Sebastian Aboul-Hassan, Jakub Marczak, Anna Szymanska, Cyprian Augustyn and Romuald Cichon. Minimally invasive thoracoscopic closure versus thoracotomy in children with patent ductus arteriosus. Journal of surgical research. 2017. 208: 1 – 9 | All patients received surgical treatment |
| 167 | 7945 | Temel MT, Coskun ME, Akbayram S, Demiryurek AT. Association between neutrophil/lymphocyte ratio with ductus arteriosus patency in preterm newborns. Brasil Med J. 2017. 118: 491 – 494 | Ductus not described as closed, but as hsPDA and without hsPDA |
| 168 | 7952 | Dany E. Weisz, Lucia Mirea, Erin Rosenberg, Maximus Jang, Linh Ly, Paige T. Church, Edmond Kelly, S. Joseph Kim, Amish Jain, Patrick J. McNamara, Prakesh S. Shah. Association of patent ductus arteriosus ligation with death or neurodevelopmental impairment among extremely preterm infants. JAMA pediatrics. 2017. 171 | Not specified when ultrasound was performed and ductus not described as closed, but as small and hsPDA |
| 169 | 7955 | Ting-An Yen, Ching-Chia Wang. Efficacy of repeated courses of ibuprofen in the closure of patent ductus arteriosus in premature infants. Pediatrics and neonatology. 2017. 58: 1 – 2 | Editorial |
| 170 | 7956 | YoungAh Youn, Cheong-Jun Moon, Jae-Young Lee, Cheul Lee, In Kyung Sung. Timing of surgical ligation and morbidities in very low birth weight infants. Medicine. 2017. 96 | Ductus not described as closed |
| 171 | 7962 | Hatice Tatar Aksoy, Nilufer Guzoglu, Zeynep Eras, Kursad Gokce, Fuat Emre Canpolat, Nurdan Uras‚ S. Suna Oguz. The association of early postnatal weight loss with outcome in extremely low birth weight infants. Pediatrics and neonatology. 2018. 1 – 5 | Not specified when ultrasound was performed and ductus not described as closed |
| 172 | 7964 | Manar Al-lawama, Iyad Alammori, Tariq Abdelghani and Eman Badran. Oral paracetamol versus oral ibuprofen for treatment of patent ductus arteriosus. Journal of international medical research. 2018. 46: 811 – 818 | Ductus not described as closed, but as not meeting inclusion criteria |
| 173 | 7968 | Vikranth Bapu Anna Venugopalan, Neil Wilson, Nick Archer, Satish Adwani. Clinical outcome at one year of age following device closure of patent ductus arteriosus (PDA) in small babies. The indian journal of pediatrics. 2018. 85: 584 | Letter to the editor |
| 174 | 7969 | Nabiollah Asadpour, Pouriya Sarboloukzadeh Harandi, Majid Hamidi, Mohammad Reza Malek Ahmadi, Afsaneh Malekpour-Tehrani. Comparison of the effect of oral acetaminophen and ibuprofen on patent ductus arteriosus closure in premature infants referred to Hajar hospital Shahrekord in 2016-2017. J clin neonatal. 7: 224 – 230 | All patients received treatment |
| 175 | 7977 | Bharathi Balachander, Nivedita Mondal, Vishnu Bhat, Bethou Adhisivam, Mahesh Kumar, Santhosh Satheesh & Mahalakshmi Thulasingam. Comparison of efficacy of oral paracetamol versus ibuprofen for PDA closure in preterms – a prospective randomized clinical trial. Journal of maternal-fetal & neonatal medicine. 2018 | Not specified when ultrasound was performed and ductus not described as closed |
| 176 | 7986 | Alona Bin-Nun, Daniel Fink, Francis B. Mimouni, Nurit Algur and Cathy Hammerman. Paracetamol Serum Concentrations in Neonates Treated Enterally for Ductal Closure: A Pilot Study. Journal of pediatrics. 2018. 198: 304 – 307 | Case report study |
| 177 | 7991 | Cristina Borràs-Novell, Ana Riverola, Victoria Aldecoa-Bilbao, Montserrat Izquierdo, Monica Domingo, Martín Iriondo. Journal de Pediatria. 2018 | Not specified when ultrasound was performed and ductus not described as closed |
| 178 | 7995 | Peter T. Cartledge, Christian Umuhoza and Catherine Harrison. In a resource-limited setting, is oral ibuprofen effective for closure of a patent ductus arteriosus in a preterm neonate? Journal of tropical pediatrics. 2016. 64: 409 – 417 | Review article |
| 179 | 8004 | Ronald I. Clyman, Melissa Liebowitz, Joseph Kaempf, Omer Erdeve, Ali Bulbul, Stellan Håkansson, Johanna Lindqvist, Aijaz Farooqi, Anup Katheria, Jason Sauberan, Jaideep Singh, Kelly Nelson, Andrea Wickremasinghe, Lawrence Dong, Denise C. Hassinger, Susan W. Aucott, Madoka Hayashi, Anne Marie Heuchan, William A. Carey, Matthew Derrick, Erika Fernandez, Meera Sankar, Tina Leone, Jorge Perez, Arturo Serize and the PDA-TOLERATE (PDA: TO LEave it alone or Respond And Treat Early) Trial Investigators. Journal of pediatrics. 2018. 1 – 8 | Not specified when ultrasound was performed and ductus not described as closed but as not hsPDA |
| 180 | 8006 | Stacey L. Crockett, Courtney D. Berger, Elaine L. Shelton, Jeff Reese. Molecular and mechanical factors contributing to ductus arteriosus patency and closure. Congenital heart disease. 2018. 1 – 6 | Overview article about pathophysiology of PDA |
| 181 | 8008 | Carlo Dani, Chiara Poggi, Ilaria Cianchi, Iuri Corsini, Venturella Vangi, Simone Pratesi. Effect on cerebral oxygenation of paracetamol for patent ductus arteriosus in preterm infants. Europena journal of pediatrics. 2018. 177: 533 – 539 | All patients received treatment |
| 182 | 8010 | Fernando de Freitas Martins, Daniel Ibarra Rios, Maura Helena F. Resende, Henna Javed, Dany Weisz, Amish Jain, Jose Maria de Andrade Lopes and Patrick J. McNamara. Relationship of patent ductus arteriosus size to echocardiographic markers of shunt volume. Journal of pediatrics. 2018. 202: 50 – 55 | Outcome too late described and PDA not described as closed |
| 183 | 8014 | Deonne Dersch-Mills, Belal Alshaikh, Amuchou S Soraisham, Albert Akierman, and Kamran Yusuf. Effectiveness of injectable ibuprofen salts and indomethacin to treat patent ductus arteriosus in preterm infants: observational cohort study. Can J Hosp Pharm. 2018. 71: 22 – 28 | All patients received treatment and not specified when ultrasound was performed |
| 184 | 8019 | Rania A. El-Farrash, Mohammed S. El Shimy, Abeer S. El-Sakka, Manal G. Ahmed & Dina G. Abdel-Moe. Efficacy and safety of oral paracetamol versus oral ibuprofen for closure of patent ductus arteriosus in preterm infants: a randomized controlled trial. Journal of maternal-fetal & neonatal medicine. 2018. 1 – 8 | Not specified when ultrasound was performed and ductus not described as closed but as hsPDAand minimal duct |
| 185 | 8025 | B Ezenwa, E Pena, A Schlegel, R Bapat, E G Shepherd, L D Nelin. Effects of practice change on outcomes of extremely preterm infants with patent ductus arteriosus. Acta paediatrica. 2018. 108: 88 – 93 | Some received prophylactic indomethacin and not specified when ultrasound was performed |
| 186 | 8027 | Daniel Fink, Afif El-Khuffash, Patrick J. McNamara, Itamar Nitzan, Cathy Hammerman. Tale of two patent ductus arteriosus severity scores: similarities and differences. Am J perinatal. 2018. 35: 55 – 58 | Comparison of two scores methods |
| 187 | 8032 | Alejandro Avila-Alvarez, Marta Serantes Lourido, Rebeca Barriga Bujan, Carolina Blanco Rodriguez, Francisco Portela-Torron, Victor Bautista-Hernandez. Surgical closure of patent ductus arteriosus in premature neonates: does the surgical technique affect the outcome? Anales de pediatria. 2017: 86: 277 - 283 | Duplicate |
| 188 | 8033 | E. Gálvez-Cuitiva, G. Lonngi-Rojas. Uso del paracetamol para el cierre del conducto arterioso en recien nacidos con edad gestacional menor a 35 semanas. Perinatologia y reproduccion humana. 2018. 32: 143 – 150 | Article not in English |
| 189 | 8034 | Jowell Garcia, Alka Garg, Yunmei Song, Ambados Fotios, Chad Andersen, Sanjay Garg. Compatibility of intravenous ibuprofen with lipids and parenteral nutrition, for use as a continuous infusion. PLoS one. 2018. 13: 1 – 13 | Pharmacological study without patients |
| 190 | 8041 | Samir Gupta, Patrick McNamara. Contemporary approach to the patent ductus arteriosus and future considerations. Semin Fetal Neonatal Med. 2018. 23: 223 – 224 | Editorial |
| 191 | 8042 | James I. Hagadorn, Mihoko V. Bennett, Elizabeth A. Brownell, Kurlen S.E. Payton, William E. Benitz and Henry C. Lee. Covariation of neonatal intensive care unit-level patent ductus arteriosus management and in-neonatal intensive care unit outcomes following preterm birth. Journal of pediatrics. 2018. 203: 225 – 233 | Not specified when ultrasound was performed |
| 192 | 8043 | H. Halil, M. Buyuktiryaki, F. Yavanoglu Atay, M. Yekta Oncel and N. Uras. Reopening of the ductus arteriosus in preterm infants: clinical aspects and subsequent consequences. Journal of neonatal-perinatal medicine. 2018. 11: 273 – 279 | Not specified when ultrasound was performed and all received treatment |
| 193 | 8044 | Pia Härkin, Riitta Marttila, Tytti Pokka, Timo Saarela & Mikko Hallman. Morbidities associated with patent ductus arteriosus in preterm infant. Nationwide cohort study. Journal of maternal-fetal & neonatal medicine. 2018. 31: 2576 – 2583 | Not specified when ultrasound was performed |
| 194 | 8045 | Sarah Louise Harris, Kiran More, Bronwyn Dixon, Richard Troughton, Chris Pemberton, John Horwood, Nicola Ellis, Nicola Austin. Factors affecting N-terminal pro-B-type natriuretic peptide levels in preterm infants and use in determination of haemodynamic significance of patent ductus arteriosus. European Journal of pediatrics. 2018. 177: 521 – 532 | Not specified when ultrasound was performed and ductus not described as closed alone but also haemodynamically non-significant |
| 195 | 8052 | Kai-Hsiang Hsu, Pierre Wong, Ram Kumar Subramanyan, Julie Evans, Shahab Noori. Predictors of respiratory improvement 1 week after ligation of patent ductus arteriosus in preterm infants. Journal of pediatrics. 2019. 205: 49 - 54 | All patients received ductal ligation and nothing reported on spontaneous closure |
| 196 | 8059 | Jihye Hwang, Yu Seon Kim, Jeong Hee Shin and Byung Min Choi. Hemodynamic effects on systematic blood flow and ductal shunting flow after loading dose of intravenous caffeine in preterm infants according to the patency of ductus arteriosus. J. Koeran Med Sci. 2018. 33: e25 | Ultrasound was performed late and the group were too old |
| 197 | 8068 | S Juujarvi, H Kallankari, P Patsi, M Leskinen, T Saarela, M Hallman, O Aikio. Follow-up study of the early, randomized paracetamol trial to preterm infants, found no adverse reactions at the two years corrected age. Acta paediatrica. 2018. 108. 452 – 458 | Follow up study |
| 298 | 8069 | S Juujarvi, T Saarela, M Hallman, O Aiki. Intravenous paracetamol was associated with closure of the ductus arteriosus in extremely premature infants. Acta paediatrica. 2018. 107. 605 – 610 | Some patients are duplicate and not specified when ultrasound was performed |
| 199 | 8072 | V. Katheria, D.M. Poeltler, M.K. Brown, K.O. Hassen, D. Patel, W. Rich, N.N. Finer and A.C. Katheria. Early prediction of a significant patent ductus arteriosus in infants < 32 weeks gestational age. Journal of neonatal-perinatal medicine. 2018. 11: 265 – 271 | Ductus not described as closed |
| 200 | 8080 | K. Kopec-Godlewsk, J.Wojkowska-Mach on behalf of the Polish Neonatology Surveillance Network Team. Infections following surgical patent ductus arteriosus ligation in very-low-birthweight neonates. Journal of hospital infection. 2018. 99: 62 – 67 | Not specified when ultrasound was performed and ductus not described as closed |
| 201 | 8087 | Jang Hoon Lee. B-type natriuretic peptide may have a role in the management of patent ductus arteriosus. Korean J Pediatr. 2018. 61: 68 – 69 | Letter to the editor |
| 202 | 8088 | David G. Lehenbauer, Charles D. Fraser III, Todd C. Crawford, Naru Hibino, Susan Aucott, Joshua C. Grimm, Nishant Patel, J. Trent Magruder, Duke E. Cameron and Luca Vricella. Surgical closure of patent ductus arteriosus in premature neonates weighing less than 1000 grams: contemporary outcomes. World journal for pediatric and congenital heart surgery. 2018. 94: 419 – 423 | All patients underwent surgical ligation |
| 203 | 8089 | Michelle B. Levinson, Catherine Messina & Jonathan P. Mintzer. Fluid management during the first postnatal day in very low birth weight neonates and rates of patent ductus arteriosus requiring treatment. Journal of maternal-fetal and neonatal medicine. 2018. 31: 2699 – 2704 | Not specified when ultrasound was performed and ductus not described as closed |
| 204 | 8093 | Deepak Louis, Cindy Wong, Xiang Y. Ye, Patrick J. McNamara & Amish Jain. Factors associated with non-response to second course indomethacin for PDA treatment in preterm neonates. Journal of maternal-fetal and neonatal medicine. 2018. 31: 1407 – 1411 | All patients had received indomethacin and no report on spontaneous closure |
| 205 | 8096 | Sally Mashally, Lynne E. Nield, Patrick J. McNamara, Fernando F. Martins, Afif El-Khuffash, Amish Jain and Dany E. Weisz. Late oral acetaminophen versus immediate surgical ligation in preterm infants with persistent large patent ductus arteriosus. J thorax Cardiovasc surg. 2018. 156: 1937 – 1944 | All patients had received treatment and no report on spontaneous closure |
| 206 | 8098 | Souvik Mitra, Ivan D. Florez, Maria E. Tamayo, Lawrence Mbuagbaw, Thuva Vanniyasingam, Areti Angeliki Veroniki, Adriana M. Zea, Yuan Zhang, Behnam Sadeghirad, Lehana Thabane. Association of placebo, indomethacin, ibuprofen and acetaminophen with closure of hemodynamically significant patent ductus arteriosus. JAMA. 2018. 319: 1221 – 1238 | Network analysis. Not specified when ultrasound was performed. |
| 207 | 8101 | Lisa Molines, Simon Nusinovici, Marie Moreau, Mathilde Remy, Pascale May-Panloup, Cyril Flamant, Jean-Christophe Roze, Patrick Van Bogaert, Pierre-Emmanuel Bouet and Géraldine Gascoin. Impact of mode of conception on neonatal and neurodevelopmental outcomes in preterm infants. Human reproduction. 2018. 1 – 9 | Follow up study. Not specified when ultrasound was performed and ductus not described as closed |
| 208 | 8102 | Patrice Morville, Stephanie Douchin, Helene Bouvaist, Claire Dauphin. Transcatheter occlusion of the patent ductus arteriosus in premature infants weighing less than 1200 g. Arch dis child fetal neonatal ed. 2018. 103: F198 – F201 | All patients had received treatment |
| 209 | 8112 | Nilüfer Okur, Cüneyt Tayman, Mehmet Büyüktiryaki Gülsüm Kadıoğlu Şimşek, Buse Ozer Bekmez, Nahide Altuğ. Can lactate levels be used as a marker of patent ductus arteriosus in preterm babies? J Clin lab anal. 2018. e22664 | Ductus not described as closed but as hsPDA and without hspDA |
| 210 | 8115 | Buse Özer Bekmez, Cüneyt Tayman, Mehmet Büyüktiryaki, Aslıhan Köse Çetinkaya, Ufuk Çakır, Turan Derme. A promising novel index om the diagnosis and follow-up of patent ductus arteriosus: red cell distribution width-to-platelet ratio. J Clin Lab Anal. 2018. 32: e22616 | Not specified when ultrasound was performed in |
| 211 | 8117 | Ozge Pamukcu, Aydin Tuncay, Nazmi Narin, Ali Baykan, Levent Korkmaz, Mustafa Argun, Abdullah Ozyurt, Suleyman Sunkak, Kazim Uzum. Patent ductus arteriosus closure in preterms less than 2 kg: surgery versus transcatheter. International journal of cardiology. 2018. 250: 110 – 115 | All patients had received treatment |
| 212 | 8122 | Sujith S. Pereira, Stephen T. Kempley, Divyen K. Shah, Joan K. Morris, Ajay K. Sinha. Early echocardiography does not predict subsequent treatment of symptomatic patent ductus arteriosus in extremely preterm infants. Acta paediatrica. 2018. 107: 1909 – 1916 | Ductus not described as closed but as without treatment |
| 213 | 8123 | Stephanie L. Perrier and Igor E. Konstantinov. Pharmacotherapy for closure of large patent ductus arteriosus in preterm neonates: fail one, not twice. J. Thorac cardiovasc surg. 2018. 156: 1935 – 1936 | Editorial |
| 214 | 8126 | Pramod Pharande, Hadley Watson, Kenneth Tan, Arvind Sehgal. Oral paracetamol for patent ductus arteriosus rescue closure. Pediatr Cardiol. 2018. 39: 183 – 190 | All patients received treatment |
| 215 | 8130 | M. Radicioni, V. Bini, G. M. Campus, P. G. Camerini. Terlipressin-induced modifications of Doppler ultrasound signals of systemic arteries in preterm infants with vasoactive-resistent patent ductus arteriosus: a pilot study. J Clin Ultrasound. 2018. 48: 202 – 208 | Case serie and not specified when ultrasound was performed |
| 216 | 8136 | Alejandro Rodrıguez Ogando, Fernando Ballesteros Tejerizo, Dorotea Blanco Bravo, Manuel Sanchez Luna and Jose Luis Zunzunegui Martıneza. Transcatheter occlusion of patent ductus arteriosus in preterm infants weighing less than 2 kg with the amplatzer duct occlude II additional sizes device. Rev Esp Cardiol. 2018. 71: 865 – 866 | All patients received treatment |
| 217 | 8137 | A. Rodrıguez Ogando, I. Planelles Asensio, A. Rodrıguez Sanchez de la Blanca, F. Ballesteros Tejerizo, M. Sanchez Luna, J. M. Gil Jaurena, C. Medrano Lopez, J. L. Zunzunegui Martınez. Surgical ligation versus percutaneous closure of patent ductus arteriosus in very low-weight preterm infants: which are the real benefits of the percutaneous approach? Pediatr cardiol. 2018. 39: 398 – 410 | All patients received treatment |
| 218 | 8139 | Silvia Rodríguez-Blanco, Ignacio Oulego-Erroz, Paula Alonso-Quintela, Sandra Terroba-Seara, Aquilina Jiménez-González, Maite Palau-Benavides. N-terminal probrain natriuretic peptide as a biomarker of moderate to severe bronchopulmonary dysplasia in preterm infants: a prospective observational study. Pediatric pulmonology. 2018. 53: 1073 – 1081 | Not known when ductus closed |
| 219 | 8145 | Hannes Sallmon, Felix Berger, Petra Koehne, Martin Koestenberger, Georg Hansmann, Bernd Opgen-Rhein. Differrnt indications for transcatheter and surgical patent ductus arteriosus closure in preterm infants less than 2 kg. International journal of cardiology. 2018. 266: 83 | Letter |
| 220 | 8146 | Hannes Sallmon, Petra Koehne. Further experience with oral paracetamol as a rescue therapy for patent ductus arteriosus in preterm infants. Pediatric cardiology. 2018. 39: 411 – 412 | Letter |
| 221 | 8147 | Hannes Sallmon, Sven C. Weber, Juliane Dirks, Tamara Schiffer, Tamara Klippstein, Anja Stein, Ursula Felderhoff-Muser, Boris Metze, Georg Hansmann, Christoph Buhrer, Malte Cremer and Petra Koehne. Association between platelet counts before and during pharmacological therapy for patent ductus arteriosus and treatment failure in preterm infants. Frontiers in pediatrics. 2018. 6, 41 | Not specified when ultrasound was performed |
| 222 | 8148 | Shyam Sathanandam, Kaitlin Balduf, Sandeep Chilakala, Kristen Washington, Kimberly Allen, Christopher Knott-Craig, Benjamin Rush Waller, Ranjit Philip. Role of transcatheter patent ductus arteriosus closure in extremely low birth weight infants. Catheter cardiovasc interv. 2018. 1 – 8 | All patients received treatment |
| 223 | 8151 | Christoph E. Schwarz, Antonio Preusche, Martin Wolf, Christian F. Poets and Axel R. Franz. Prospective observational study on assessing the hemodynamic relevance of patent ductus arteriosus with frequency domain near-infrared spectroscopy. BMC pediatrics. 2018. 18: 66 | Not specified when ultrasounds was performed and ductus not described as closed but as hrPDA or no-hrPDA |
| 224 | 8168 | Tomasz Stankowski, Sleiman Sebastian Aboul-Hassan, Dirk Fritzsche, Marcin Misterski, Jakub Marczak, Anna Szymanska, Lukasz Szarpak, Cyprian Augustyn, Romuald Cichon, Bartlomiej Perek. Surgical closure of patent ductus arteriosus in extremely low birth weight infants weighing less than 750 grams. Kardiologia Polska. 2018. 76: 750 – 754 | All patients received treatment |
| 225 | 8169 | Robin H. Steinhorn. Trends in PDA treatment: is less really more? Journal of pediatrics. 2018. 203: 2 | No patients involved |
| 226 | 8172 | Jane E. Stremming, Clyde J. Wright, Laura D. Brown. Is paracetamol as effective as indomethacin or ibuprofen in closing a hemodynamically significant patent ductus arteriosus in preterm infants? Acta paediatrica. 2018. 107: 1836 | Commentary on article |
| 227 | 8175 | Elizabeth J. Thompson, Rachel G. Greenberg, Karan Kumar, Matthew Laughon, P. Brian Smith, Reese H. Clark, Andromeda Crowell, Layla Shaw, Louis Harrison, Gabrielle Scales, Nicole Bell and Christoph P. Hornik. Association between furosemide exposure and patent ductus arteriosus in hospitalized infants of very low birth weight. | Not specified when ultrasounds was performed and ductus not described as closed but as PDA or no PDA |
| 228 | 8180 | Danitza M. Velazquez, Kimberly J. Reidy, Madhu Sharma, Mimi Kim, Melissa Vega & Tomas Havranek. The effect of hemodynamically significant patent ductus arteriosus on acute kidney injury and systemic hypertension in extremely low gestational age newborns. Journal of maternal-fetal and neonatal medicine. 2018. 1 – 6 | Ductus not described as closed but as hsPDA or no hsPDA |
| 229 | 8184 | Dany E. Weisz, Lucia Mirea, Maura H.F. Resende, Linh Ly, Paige T. Church, Edmond Kelly, S. Joseph Kim, Amish Jain, Patrick J. McNamara and Prakesh S. Shah. Outcomes of surgical ligation after unsuccessful pharmacotherapy for patent ductus arteriosus in neonates born extremely preterm. Journal of pediatrics. 2018. 195: 292 – 296 | All patients received treatment |
| 230 | 8185 | Kent A. Willis, Mark F. Weems. Hemodynamically significant patent ductus arteriosus and the development of bronchopulmonary dysplasia. Congenital heart disease. 2018. 1 – 6 | Overview article, no patients included |
| 231 | 8187 | Jonathan P. Wyllie, Samir Gupta. Prophylactic and early targeted treatment of patent ductus arteriosus. Seminars in fetal and neonatal medicine. 2018. 23: 250 – 254 | Overview article, no patients included |
| 232 | 8191 | Sadık Yurttutan, Aydın Bozkaya, Füheda Hüdayioglu & Mehmet Yekta Oncel. The effect of combined therapy for treatment of monotherapy-resistant PDA in preterm infants. Journal of maternal-fetal and neonatal medicine. 2018. 1 – 4 | Case serie report. All received treatment |
| 233 | 8194 | Hitesh Agrawal, Benjamin Rush Waller III, Sushitha Surendan, Shyam Sathanandam. New patent ductus arteriosus closure devices and techniques. Intervent. Cardiol clin. 2019. 8: 23 – 32 | Review of devices for transcatheter closure |
