# Supplementary material for: Spontaneous Closure of the Ductus Arteriosus in Preterm Infants: A Systematic Review
Source: Front Pediatr. 2020 Sep 11;8:541. doi: 10.3389/fped.2020.00541 (PMC7516116; doi:10.3389/fped.2020.00541)
Supplement: Supplementary file 3 [file Table_3.docx]

Supplementary File 3. Summary of the clinical characteristics of the included studies.

| Ref no. | Ref no. endnote | Group | Author | Year of publication | Total eligible infants | Inclusion criteria | | GA at birth | BW at birth | Ultrasound PDA (hours) |
| --- | --- | --- | --- | --- | --- | --- | --- | --- | --- | --- |
|  |  |  |  |  |  | GA | BW | Mean (SD)/Median (IQR) | Mean (SD)/Median (IQR) |  |
| 98 | 544 | 4 | D.B. Shortland | 1990 | 104 | <33 | <1501 | 29 (28 – 32) | 1140 and 1240 | 24, 48, 72, 96, 120, 144, 168 |
| 26 | 1657 | 2 | M.D. Reller | 1991 | 26 | <30 |  | 28,8 +/- 1,4 | 1259 +/- 332 | 10,6; 32,8; 56,7; 79,8 |
| 37 | 1925 | 2 | L.R. Ment | 1994 | 431 |  | <1250 | 28 +/- 2,2 | 947 +/- 176 | 24; 120 |
| 45 | 138 | 3 | R. Skelton | 1994 | 55 | <31 | <1500 | 27 (25 – 31) | 1000 (512 – 1500) | 24; 48; 72; 96; 120; 144; 168 |
| 113 | 482 | 4 | B. van Overmeire | 1995 | 220 | <34 |  | <34 |  | 48 – 96 |
| 102 | 1029 | 4 | B. van Overmeire | 1997 | 85 | <33 |  | <33 |  | 48 – 72 |
| 46 | 41 | 3 | B.H. Su | 1997 | 37 |  | <1500 | 29,2 +/- 2,5 | 1132 +/- 260 | 24; 48; 96; 120 |
| 57 | 1013 | 3 | M.P. De Carolis | 2000 | 50 | <31 |  | 28 +/- 1,9 | 993 +/- 308 | 72 |
| 15 | 1514 | 1 | M. Narayanan | 2000 | 170 | <28 |  | 25,5 +/- 1,1 | 803 +/- 180 | 72 |
| 68 | 15 | 3 | D. Trevisanuto | 2000 | 23 | <32 |  | 27,5 +/- 0,8 | 891 +/- 120 | 48 |
| 79 | 1230 | 3 | R. Arlettaz | 2001 | 83 | <32 |  | <32 | <1500 | 48 – 144; 120 |
| 89 | 922 | 3 | H. Suzumura | 2001 | 138 | <32 |  | 28,4 (23 – 31) | 1193 (455 – 1695) | 168 |
| 90 | 88 | 3 | B. van Overmeire | 2001 | 63 | <32 |  | 29,2 +/- 2,1 | 1270 +/- 365 | 144; 216 |
| 97 | 485 | 4 | P. Lago | 2002 | 232 | <34 |  | <34 |  | 48 – 72 |
| 111 | 254 | 4 | V.F. Puddy | 2002 | 18 | <34 |  | 30 (24 – 34) | 1460 (540 – 2130) | 168 |
| 91 | 309 | 3 | E. Heyman | 2003 | 52 | <32 | <1500 | <32 | <1500 | 48 – 96 |
| 16 | 43 | 1 | V. Gournay | 2004 | 66 | <28 |  | 26 +/- 0,9 | 851 +/- 164 | 72 |
| 92 | 275 | 3 | L. Schmitz | 2004 | 22 |  | <1500 | 28,6 +/- 2 | 987 +/- 248 | 86,4; 338 |
| 38 | 446 | 2 | B. van Overmeire | 2004 | 415 | <30 |  | 28,1 +/- 1,6 | 1065 +/- 324 | 6; 72 |
| 112 | 256 | 4 | M.C. Byung | 2005 | 66 | <34 |  | 30,3 +/- 2,4 | 1396 +/- 418 | 72, 120 |
| 18 | 1818 | 1 | C. Dani | 2005 | 155 | <28 |  | 25,9 +/- 1,1 | 812 +/- 209 | 24; 72; 120; 168 |
| 93 | 199 | 3 | S. Sanjeev | 2005 | 29 |  | <1500 | 26 +/- 2 | 873,5 +/- 247 | 24 |
| 47 | 784 | 3 | J. Koch | 2006 | 122 | <32 | <1000 | 26 +/- 1,9 | 794 +/- 118 | 103,2 |
| 48 | 137 | 3 | T. Rakza | 2007 | 48 | <32 |  | 29 +/- 1,4 | 1300 +/- 160 | 6 |
| 49 | 600 | 3 | A. El-Khuffash | 2008 | 80 | <32 | <1500 | 28,6 (24,1 – 31,6) | 1080 (600 – 1500) | 48 |
| 50 | 1002 | 3 | A. El-Khuffash | 2008 | 33 |  | <1500 | 28,7 (24,1 – 31,1) | 1110 (670 – 1420) | 72 |
| 39 | 3768 | 2 | A.M. Groves | 2008 | 80 | <30 |  | 28 (24 – 30) | 1060 (510 – 1900) | 5; 12; 24; 48 |
| 51 | 1808 | 3 | S.L. Nemerofsky | 2008 | 65 |  | <1500 | 27,5 +/- 2,2 | 1019 +/- 258 | 72; 168; discharge |
| 52 | 1139 | 3 | C. Sangtawesin | 2008 | 62 |  | <1500 | 29,29 +/- 2,16 | 1162,9 +/- 261 | 72 |
| 19 | 172 | 1 | B.H. Su | 2008 | 143 | <28 |  | <28 |  | 12 – 24 |
| 11 | 141 | 2 | J.V. Aranda | 2009 | 136 | <30 | <1000 | 26,2 +/- 1,4 | 797,3 +/- 132,8 | 336 |
| 106 | 119 | 4 | P. Nuntnarumit | 2009 | 35 | <33 |  | 31 (28 – 33) | 1360 (731 – 1831) | 48, 96 |
| 53 | 1752 | 3 | R. Vieux | 2010 | 74 | <32 |  | 28,3 +/- 1,2 | 1108,1 +/- 287 | 48; 168 |
| 54 | 56 | 3 | T. Gokmen | 2011 | 353 | <32 |  | <32 | <1500 | 72 |
| 40 | 1032 | 2 | V.K. Kalra | 2011 | 52 |  | <1250 | <34 | <1250 | 72 – 168 |
| 96 | 39 | 4 | A. Khositseth | 2011 | 58 | <33 |  | 30,8 +/- 1,3 | 1543 +/- 361 | 45,4 |
| 55 | 268 | 3 | D. Martinovici | 2011 | 31 | <32 | <1500 | 29,8 +/- 2,2 | 1290 +/- 360 | 96; 168 |
| 101 | 761 | 4 | P. Nuntnarumit | 2011 | 50 | <33 |  | 31 (29 – 31) | 1640 (925 – 2060) | 48, 168 |
| 56 | 1098 | 3 | T. Strauss | 2011 | 120 | <32 |  | 28,1 +/- 2,3 | 1037 +/- 370 | 115,2 |
| 60 | 104 | 3 | A. Dizdar | 2012 | 361 | <32 |  | 29 (27 – 30) | 1050 (880 – 1290) | 72 – 120 |
| 58 | 180 | 3 | S. BUddhe | 2012 | 69 |  | <1500 | 28 +/- 2,4 | 1053 +/- 278 | 352,8 |
| 59 | 657 | 3 | R. Desandes | 2012 | 252 | <32 |  | 29 +/- 1,4 | 1249 +/- 311 | 45,6 |
| 20 | 560 | 1 | O. Erdeve | 2012 | 183 | <28 | <1000 | <28 | <1000 | 72 |
| 61 | 201 | 3 | H. Sallmon | 2012 | 1350 |  | <1500 | 29 6/7 (22 – 37 3/7) | 1230 (356 – 1495) | 96 – 120 |
| 41 | 999 | 2 | S. Alan | 2013 | 46 | <30 |  | 28 +/- 1,7 | 1042 +/- 200 | 24 – 48 |
| 42 | 267 | 2 | R. Braulio | 2013 | 215 | <30 |  | <30 |  | 72 – 96 |
| 62 | 264 | 3 | B. Brunner | 2013 | 322 | <32 | <1500 | 29 (23 – 31) | 1190 (420 – 1500) | 48 |
| 21 | 924 | 1 | J. candel-Pau | 2013 | 65 | <28 |  | 25,9 +/- 1,37 | 840 +/- 206,3 | 48 |
| 43 | 925 | 2 | C. Dani | 2013 | 163 | <30 |  | 27,5 +/- 1,1 | 985 +/- 225 | 12 – 24 |
| 109 | 166 | 4 | A. Khositseth | 2013 | 30 | <34 |  | 31 (29 – 32) | 1490 (1100 – 1670) | 24, 72, 1632 |
| 108 | 128 | 4 | K. Mine | 2013 | 46 | <33 | <1500 | 28,1 (25,5 – 29,2) | 950 (799 – 1180) | 48 |
| 63 | 1012 | 3 | A. Sellmer | 2013 | 183 | <32 |  | 29 (24 – 31) | 1190 (470 – 2160) | 72 |
| 64 | 1629 | 3 | L. Visconti | 2013 | 119 |  | <1500 |  | <1500 | 324 |
| 110 | 241 | 4 | Y.J. Ding | 2014 | 72 |  |  | 30,24 +/- 1,49 | 1468,64 +/- 447,62 | 168 |
| 65 | 959 | 3 | B. Grass | 2014 | 52 | <32 |  | 29,2 (26,9 – 31,3) | 1160 (910 – 1600) | 48; 144 |
| 100 | 732 | 4 | A.J. Khosroshahi | 2014 | 60 | <34 | <2500 | 31 +/- 1,9 | 1680 +/- 350 | 70,32 |
| 66 | 944 | 3 | J.B. Letshwiti | 2014 | 69 |  | <1500 | 28,6 +/- 2,6 | 1119 +/- 257 | 168 |
| 67 | 685 | 3 | F. Occhipinti | 2014 | 41 | <32 |  | 29,8 +/- 2,4 | 1309 +/- 464 | 6 – 24 |
| 44 | 130 | 2 | M.Y. Oncel | 2014 | 228 | <30 | <1250 | <30 | <1250 | 72 |
| 27 | 94 | 2 | R. Parikh | 2014 | 64 | <30 |  | 27,1 | 940 | 72 |
| 22 | 505 | 1 | A. Rolland | 2014 | 91 | <28 |  | 26,3 +/- 1 | 823 +/- 164 | 72; 1464 |
| 107 | 123 | 4 | D. Terek | 2014 | 292 | <34 |  | 31,04 +/- 2,3 | 1669 +/- 531 | 48 – 72 |
| 69 | 76 | 3 | H.L. Chen | 2015 | 52 | <32 | <1500 | 28,6 +/- 1,6 | 1120 +/- 260 | 168; 3864 |
| 70 | 65 | 3 | K. Konig | 2015 | 58 | <32 |  | 27 3/7 +/- 2 2/7 | 1032 +/- 315 | 32,9 |
| 23 | 1970 | 1 | M. Steiner | 2015 | 104 | <28 |  | 26 +/- 1,4 | 807 +/- 205 | 48 – 144 |
| 71 | 206 | 3 | M. Akar | 2016 | 353 | <32 | <1500 | <32 | <1500 | 48 – 96 |
| 28 | 394 | 2 | A. Bin-Nun | 2016 | 101 | <30 |  | 28,1 +/- 1,7 | 1098 +/- 249 | 72 – 96 |
| 73 | 1058 | 3 | N. Demir | 2016 | 243 | <32 |  | 29 (26 – 32) | 1595 (640 – 2000) | 72 – 120 |
| 74 | 74 | 3 | L. Dix | 2016 | 380 | <32 |  | <32 |  | 48 |
| 75 | 5342 | 3 | Y. Elsayed | 2016 | 71 | <31 |  | 28 +/- 1,6 | 1101 +/- 280 | 48 – 72 |
| 76 | 467 | 3 | D. Engur | 2016 | 53 | <32 |  | 30 (29 – 31) | 1410 (1112 – 1709) | 120 – 168 |
| 77 | 721 | 3 | P. Harkin | 2016 | 25 | <32 |  | 28,3 +/- 2,06 | 1120 +/- 340 | 24; 48; 72; 96; 120 |
| 99 | 594 | 4 | V.V. Kulkarni | 2016 | 80 | <34 |  | <34 |  | 72 |
| 78 | 5422 | 3 | Mochammading | 2016 | 33 | <32 | <1500 | 31 (28 – 31) | 1361 (1000 – 1500) | 48 – 72; 120 – 168; 240 |
| 80 | 5343 | 3 | A. Oliveira | 2016 | 328 | <32 | <1500 | 31 (23 – 36) | 1280 (520 – 2210) | 24 – 72 |
| 24 | 3 | 1 | T.B. Polat | 2016 | 58 | <28 | <1000 | 26, 6 +/- 1,1 | 824 +/- 131 | 76,4 |
| 81 | 698 | 3 | K.A. Tauber | 2016 | 95 | <32 |  | 30 +/- 1 5/7 | 1371 +/- 443 | 120 |
| 82 | 163 | 3 | E. Valerio | 2016 | 196 | <32 |  | 23 – 32 |  | 48 – 72 |
| 83 | 193 | 3 | M.E. van der Laan | 2016 | 49 | <32 |  | 27,6 (26,1 – 29) | 980 (800 – 1200) | 77 (70 – 107) |
| 29 | 7806 | 2 | C.R. Breatnach | 2017 | 121 | <30 |  | 26,8 +/- 1,4 | 968 +/- 250 | 10 (7 – 13); 43 (38 – 46); 143 (125 – 161) |
| 72 | 2555 | 3 | C.R. Breatnach | 2017 | 51 | <32 |  | 27 (25,1 - 30,7) | 1060 (750 – 1360) | 10; 42; 143 |
| 30 | 7817 | 2 | K. de Waal | 2017 | 25 | <30 |  | 28 (25 – 29) | 1062 (630 – 1530) | 72; 168; 336 |
| 84 | 7818 | 3 | N. Demir | 2017 | 212 | <32 | <1500 | <32 | <1500 | 72 |
| 31 | 7829 | 2 | Y. Elsayed | 2017 | 70 | <30 |  | 28 +/- 1,6 | 1101 +/- 280 | 48 – 72 |
| 32 | 7844 | 2 | E. Gomez-Pomar | 2017 | 31 | <30 |  | <29 |  | 192 |
| 85 | 7879 | 3 | A. Ledo | 2017 | 72 | <32 |  | <32 |  | 36 – 48 |
| 25 | 7881 | 1 | J.H. Lee | 2017 | 15971 | <28 |  | <28 | <1500 | 48 – 144 |
| 17 | 7885 | 1 | M. Liebowitz | 2017 | 150 | <28 |  | 26 +/- 1,2 | 802 +/- 200 | 168 |
| 95 | 78 | 4 | O. Olukman | 2017 | 824 | <34 |  | 24 – 34 |  | 96 |
| 86 | 7931 | 3 | A. Sellmer | 2017 | 146 | <32 |  | <32 |  | 72 |
| 103 | 7974 | 4 | H. Babaei | 2018 | 33 | <34 |  | 31,2 +/- 1,8 | 1917 +/- 429,6 | 72 |
| 104 | 7976 | 4 | M.M. Bagheri | 2018 | 80 | <34 |  | 30,38 +/- 1,44 | 1355,25 +/- 245,92 | 72 |
| 33 | 8011 | 2 | K. de Waal | 2018 | 48 | <30 |  | 24 – 29 | 610 – 1260 | 72; 672 |
| 87 | 8013 | 3 | G. Demirel | 2018 | 100 | <32 | <1500 | 28,5 +/- 2 | 1241 +/- 432 | 72 – 120 |
| 105 | 8016 | 4 | Y. Ding | 2018 | 68 | <36 |  | 30,21 +/- 1,96 | 1479,19 +/- 474,80 | 72, 168 |
| 34 | 8070 | 2 | D. Kahvecioglu | 2018 | 60 | <30 |  | 27,8 +/- 1,7 | 1024 +/- 285 | 72 – 96 |
| 36 | 8082 | 2 | M. Kucuk | 2018 | 75 | <32 |  | 28,5 +/- 2,4 | 1180 +/- 406 | 12 – 24 |
| 35 | 8141 | 2 | V. Romagnoli | 2018 | 593 | <30 |  | 28,7 +/- 2,4 | 1028 +/- 238 | 48 – 72 |
| 88 | 8190 | 2 | S.K. Yum | 2018 | 71 |  |  | 28,7 (27,8 – 30) | 1109 (966 – 1331) | 72; 168 +/- 24; 336 +/- 48; 672 +/- 72 |
